# Perceived barriers and facilitators of accessing statutory and non-statutory services, in disadvantaged communities, in England: a co-produced qualitative review

**DOI:** 10.3389/phrs.2026.1608969

**Published:** 2026-05-28

**Authors:** Kristof Santa, Rosie Gordon, Buket Kara, Jorg Huber, Henry Pollock, Heather Catt, Prabhad Jayakody, Koser Khan, Andrew Harding, Anna Kenyon, Jade Swain-Veneziale, Amalia Theophilou, Grace Robson, Barbara Mezes

**Affiliations:** 1 University of Liverpool, Liverpool, United Kingdom; 2 The University of Manchester, Manchester, United Kingdom; 3 Lancaster University, Lancaster, United Kingdom; 4 University of Brighton, Brighton, United Kingdom; 5 BoingBoing Foundation, Blackpool, United Kingdom; 6 NerdyCatt Ltd., Preston, United Kingdom; 7 Blackpool Teaching Hospitals NHS Foundation Trust, Blackpool, United Kingdom; 8 University of Lancashire, Preston, United Kingdom; 9 Blackpool Council, Blackpool, United Kingdom; 10 Inspiring Me to Dream Ltd, Preston, United Kingdom

**Keywords:** community health services, co-produced review, disadvantaged populations, health services accessibility, qualitative research

## Abstract

**Objectives:**

This review explored the perceived barriers and facilitators to accessing statutory and non-statutory health and social care services among disadvantaged populations in England. Community-based services delivered by statutory bodies and the voluntary, community, faith, and social enterprise (VCSFE) sector are critical for addressing social determinants of health, fostering resilience, and promoting equity. However, in disadvantaged areas, complex needs and structural exclusion frequently limit their effectiveness.

**Methods:**

Co-produced with academics, VCFSE sector, and public contributors, this review synthesised qualitative research published since 2003, drawing on first-hand accounts of service users to explore how intersecting forms of disadvantage affect service access.

**Results:**

The review identified four themes influencing access to community-based services among disadvantaged groups: structural and informational barriers, the impact of cultural, social, and economic contexts, institutional trust and continuity, and emotional motivators and barriers.

**Conclusion:**

Improving access to community-based services requires structural and cultural alignment with users’ lives. Services ought to prioritise flexibility, trust, and navigational support while addressing emotional and institutional barriers.

## Introduction

Community-based statutory and non-statutory services play a vital role in addressing social determinants of health and promoting equity, especially in disadvantaged communities. In England, statutory services are provided or commissioned by public bodies, e.g., local authorities, National Health Service (NHS), as required by law [1]. Non-statutory services are delivered by a range of providers, including voluntary, community, faith, and social enterprise (VCFSE) organisations, private sector partners, and in some cases, local authorities, depending on local needs and resources [2]. These services promote social cohesion [3], foster resilience [4], and support personal growth [5], through local events, community centres [6], and outreach efforts that enhance connection [7] and belonging [8]. Internationally, evidence increasingly supports community-centred and integrated approaches to care, as well as practical strategies such as community health programmes, outreach/mobile services, telemedicine, and partnership with voluntary and faith-based organisations, as important frameworks for improving access and addressing inequities in underserved communities [9]. The World Health Organization has emphasised that reducing health inequities requires community-centred, integrated services that are responsive to local social and economic conditions [10]. A systematic review suggests that better system navigation and community-linked models can improve access to health and social care for underserved populations by reducing fragmentation and helping people engage with services more effectively [11]. In addition, evidence from both high-income and low- and middle-income countries indicates that community health worker and outreach-based approaches can strengthen trust and equitable access via locally responsive efforts [12]. Culturally responsive services were also shown to improve access and foster resilience [13, 14], supporting health through education, mental healthcare, and cultural activities [15].

Despite their importance, access to community-based services remains unequal. Intersectionality and health equity frameworks are critical for understanding the experiences of communities facing multiple, overlapping forms of systemic disadvantage [16]. Intersectionality, as articulated by Crenshaw, highlights how social identities such as race, gender, socioeconomic status, disability, and migration status interact to produce unique forms of marginalisation that cannot be understood in isolation [17]. Health equity theory extends this by emphasising the need to address the structural and social determinants of health, such as access to care, discrimination, and resource distribution, that systematically disadvantage certain groups [18]. Levesque et al.’s patient-centred access framework complements these perspectives by conceptualising access as a relationship between health-system characteristics and people’s abilities to identify need, seek, reach, afford, and engage with care across the care pathway [19]. This framing is relevant in contexts of intersectional health equity because it situates barriers within the fit between services and people’s lived circumstances [20], and helps explain how disadvantaged groups can face overlapping linguistic, administrative, financial, and trust-related barriers even where services formally exist [21]. Service access is also a negotiated process, as the Candidacy theory highlighted [22], through whether individuals recognise themselves, and are recognised by services, as legitimate candidates for care [23]. This negotiation occurs as people interpret their needs, find routes into care, present concerns in ways that services can act on, and have those concerns assessed through professional judgement [24]. Therefore, the institutional perspective of entering and remaining in care is represented by whether service users’ needs are recognised at all, delayed, or left unmet [25]. Together, these perspectives underscore that health inequalities are shaped by intersecting systems of power and inequity [26].

For communities experiencing multiple layers of disadvantage, this means that barriers to health and wellbeing are compounded, requiring responses that are not only inclusive but also structurally informed, culturally responsive, and attentive to the complexity of lived experiences [17]. Disadvantaged populations in England, such as those living in poverty, ethnic minority groups, migrants, LGBTQIA+ individuals, disabled people, and those experiencing homelessness [27], face persistent and complex barriers when compared to those in affluent regions [28, 29]. For example, LGBTQIA+ individuals and people with disabilities often face additional barriers to support [30], including discrimination, inaccessible formats [31], and exclusion from programmes. These are often the very groups most in need of services, yet they experience higher rates of unmet needs [32], delayed care [33], and social isolation [34]. Furthermore, reduced funding, staff shortages, and poor infrastructure limit service availability and quality, undermining equity goals [35, 36]. Low-income individuals may be unable to afford travel [37], while rural and remote areas face long distances and fragmented services [38, 39]. Cultural and linguistic mismatches also hinder access for ethnic minority groups, with miscommunication, bias, and limited cultural competence reducing engagement [30, 40]. However, evidence from South Korea [41] and Canada [42] shows that culturally competent, co-designed services build trust and improve uptake.

Research to date has identified several factors that can facilitate service access for disadvantaged communities [9]. For instance, community outreach and health education can build trust and awareness [43, 44], which is particularly important for disadvantaged populations who may have low trust in formal services, low awareness of entitlements, language barriers, and limited confidence in engaging with formal services [45]. When delivered with community leaders and local organisations [46], these approaches can reduce social distance from services and improve engagement among socially excluded groups, although a recent review suggests that their effectiveness depends on sustained relationships and links with mainstream provision [45]. These models have also, shown success in high-income countries like the USA [47] and in LMICs (e.g., Zimbabwe [48]), where community health workers bridge service gaps for underserved populations by supporting advocacy and service navigation [42], which have particular importance for disadvantaged groups with multiple and intersecting needs [49]. Integrated care models can reduce fragmentation and improve user experience, as shown internationally [50] and increasingly in the UK [51], helping people navigate services and improving continuity [52]. Social networks, including peer support and volunteer schemes, were found to offer advocacy and emotional support, facilitating access [53, 54]. Furthermore, health information technologies (HITs) have been found to help overcome logistical barriers [55], reduce missed appointments, and improve uptake [56]. Designing these systems inclusively, with attention to inequalities in digital literacy and access across age, income, and ability [57], can increase responsiveness and user satisfaction [58], as digital tools can otherwise reproduce existing inequalities in these domains [59].

However, important gaps remain in the evidence base. While existing research has identified key barriers and facilitators to service access, there is no comprehensive review of how disadvantaged populations experience these factors [60, 61], nor sufficient analysis of how intersecting marginalisations, e.g., minority ethnicity, disability, sexual and gender identity, and poverty, compound access barriers [62–64]. Additionally, the UK literature is heavily skewed toward quantitative studies, with limited qualitative research exploring the lived experiences of disadvantaged populations [61, 65]. This is particularly relevant, as policy and practice increasingly value user voice and community involvement in shaping sustainable services [66–68]. Addressing these gaps requires the inclusion of diverse user perspectives and methodological pluralism to inform the development of more equitable service systems. Consequently, this co-produced qualitative review aimed to synthesise qualitative evidence on the perceived barriers and facilitators of accessing statutory and non-statutory health and social care services among disadvantaged communities in England.

## Methods

### Co-production

The review question, methodology, and final manuscript check were co-produced through the “Community Solutions for Health Equity (CSfHE) project”, based in a socioeconomically disadvantaged area in England. Non-academic co-researchers led the identification of the research question and the design of the review. The data analysis involved academic co-researchers; however, non-academic co-researchers participated in a consultative capacity and were invited to review the identified themes, as well as their structure and interpretation to generate the final themes. Non-academic co-researchers shaped the interpretation of themes by reviewing the written interpretation of all themes and subthemes, providing comments and suggestions on their accuracy, relevance, and resonance with community and practice-based understandings of service access. These comments were incorporated into the revised theme interpretations, after which co-researchers reviewed the amended version again to confirm that the final interpretation was accurate and relevant. There were no disagreements in the interpretation of themes. Comments were made on a shared document, which enabled contributors to review the material asynchronously and suggest changes directly to the manuscript. Although academic co-researchers retained responsibility for coordinating the manuscript, the use of a shared document supported transparent decision-making by making comments, responses, and revisions visible to all contributors. The collaboration followed INVOLVE’s principles [69] and included co-researchers from the VCFSE sectors and academia, and public representatives, over three sessions. Nineteen initial review questions were refined using the PICO framework [70], prioritised, and discussed with the co-production group, resulting in nine refined questions. The highest-priority question, chosen based on the group’s regional experience, informed this review: “What are the perceived barriers and facilitators of accessing statutory and non-statutory services, in disadvantaged communities?”

### Design

The review protocol was pre-registered via PROSPERO (Reference No. CRD42024546994). Also, the review used the thematic synthesis approach, to integrate and analyse findings from included studies [71]. Reporting followed ENTREQ guidelines for qualitative synthesis [72] and PRISMA guidelines [73]. This review was informed by a health equity perspective, with a focus on how overlapping forms of disadvantage may shape experiences of service access. The review sought to interpret service users’ accounts in relation to the structural, relational, cultural, and emotional conditions affecting engagement.

### Search strategy

The search strategy was iteratively developed. KS created initial terms, with input from a university librarian. Specific and truncated phrases were derived from common literature expressions. The research team, then, refined and finalised the search strategy.

Four main concepts guided the search:

- service use OR access OR barrier OR facilitator
- AND community-based statutory service OR non-statutory service
- AND England
- AND disadvantaged

Relevant synonyms were used across all databases (see Supplementary Material 1 for details). MeSH terms were excluded due to relevance concerns and indexing delays. The databases searched were, PubMed (including Medline), Scopus, APA PsycInfo, and Web of Science Core Collection. Limits included publication date, English language, and publication type. Additional records were identified via handsearching strategies, including reference checking and supplementary searches in Google Scholar, Semantic Scholar, and Connected papers, with all retrieved records screened against the same eligibility criteria. The search concluded on 18 June 2024.

### Eligibility criteria

Articles before 2003 were excluded due to the NHS Reform and Healthcare Professions Act 2002, which likely affected service accessibility in disadvantaged areas [74] (Table 1). Only qualitative studies exploring service user experiences in England were included, given differences within health systems in the United Kingdom (UK) [75, 76]. Non-English publications, grey literature, opinion pieces, and secondary interpretations were excluded. Primary care studies were included, even when linked to secondary or tertiary care (e.g., maternity services) [77, 78]. Included studies had to focus on disadvantaged populations (e.g., due to socioeconomic status, race/ethnicity, sexual or gender identity, disability) [27]. The review only included direct, primary-source accounts of service users, to maintain focus on lived experiences of target groups [79].

**TABLE 1:** Inclusion and exclusion criteria (England, 2003–2024).

​	Inclusion criteria	Exclusion criteria
Publication date	Articles published after January 2003	Articles published before January 2003
Study methodology	The articles were peer-reviewed and used qualitative or mixed methods with a qualitative component The qualitative findings included first-hand accounts from service users	Quantitative studies without a qualitative component, case studies, opinion pieces, conference abstracts, grey literature, or study protocols Reviews and studies that synthesised existing literature without providing primary qualitative data
Setting and context	The study was conducted in England The research focused on community-based primary health and social care services. This may include secondary or tertiary services (e.g., maternity services), if they addressed only primary care settings Research must address a specific service or specific type of service, such as substance misuse services, mental health services, or other community-based interventions	The study was not conducted in England The research did not focus on primary care settings via community-based health and social care services The study was solely concerned with COVID-19 conditions
Population	The study focused on disadvantaged populations in England Community leaders or community champions were included if they were part of the disadvantaged population being studied and contributed with insights in relation to service access Studies that included mixed populations (disadvantaged and non-disadvantaged populations from England and other countries) were included, if the findings were separated, and the insights from the disadvantaged population(s) in England could be clearly distinguished The population could include any age group if they met the disadvantaged criterion	The study that did not involve a disadvantaged population The study involved mixed populations (disadvantaged and non-disadvantaged or populations from different countries) without separating the findings and not allowing for independent analysis of the disadvantaged group
Condition/ Content	The study focused on barriers and facilitators to service access, particularly for primary care in community-based services The qualitative findings came from primary sources, such as interviews, workshops, co-production groups, or focus groups with service users	The study explored experiences of service access in general but failed to focus on specific services or interventions The study focused solely on service providers` insights, and did not provide first-hand accounts of service access experience

### Study selection

Two authors conducted a single-blind screening. KS screened all articles, RG screened 20%. Inter-rater reliability (Cohen’s Kappa) was *κ* = 0.813 (*SE* = 0.044), indicating almost perfect agreement [80]. KS screened all full texts, RG screened 20%. Inter-rater reliability was *κ* = 0.726 (*SE* = 0.064), indicating substantial agreement. All disagreements were resolved through discussions between the two reviewers, and no third reviewer was required given the level of agreement observed.

### Data extraction

Prior to extraction, procedures were piloted. KS and PJ extracted qualitative primary source data from service users in relation to barriers and facilitators of service access into an Excel database. RG checked 20% of entries for accuracy and completeness. Data from each included article were reviewed and extracted under the relevant headings in the extraction form.

### Quality appraisal

The Critical Appraisal Skills Programme (CASP) Checklist for Qualitative Research [81] was used to appraise the methodological quality and rigor of the studies. KS conducted the appraisal, with 20% checked by RG. A Kappa value of 0.750 (*SE* = 0.043) indicated substantial agreement. All disagreements were resolved through discussions. No articles were excluded based on quality. The quality assessments are presented in Supplementary Table 2.

### Data analysis

This review followed a thematic synthesis approach [71]. NVivo 12 was used to manage and code extracted qualitative data. The process involved three key steps: [1] line-by-line coding of service user data to capture initial concepts, [2] development of descriptive themes by grouping similar codes, and [3] generation of analytical themes that offered broader interpretations grounded in the data. KS led the analysis, with BM and BK reviewing the coding and interpretations. While data extraction was deductive (focusing on barriers and facilitators), theme development was inductive. Interpretation was informed by a health equity perspective, with attention to intersectionality and to how overlapping forms of disadvantage and exclusion shaped experiences of service access across studies. Reflexive practices were maintained throughout to mitigate bias and ensure analytical rigour.

### Descriptive comparison of subtheme representation by study period

Studies were grouped into pre-COVID (n = 34) and during and post-COVID (n = 8) based on the year of data collection. This division was used because the pandemic substantially altered the organisation and delivery of health and social care services, including the disruption to face-to-face provision and increased socioeconomic pressures affecting service engagement. There was no distinction made between during and post-COVID, because these studies did not specify whether their data collection took place during or post-COVID. Supplementary Table 3 was used to descriptively compare the representation of themes and subthemes across these periods, which was intended to identify broad patterns in subtheme representation across studies.

## Results

### Identification of studies

**FIGURE 1:**
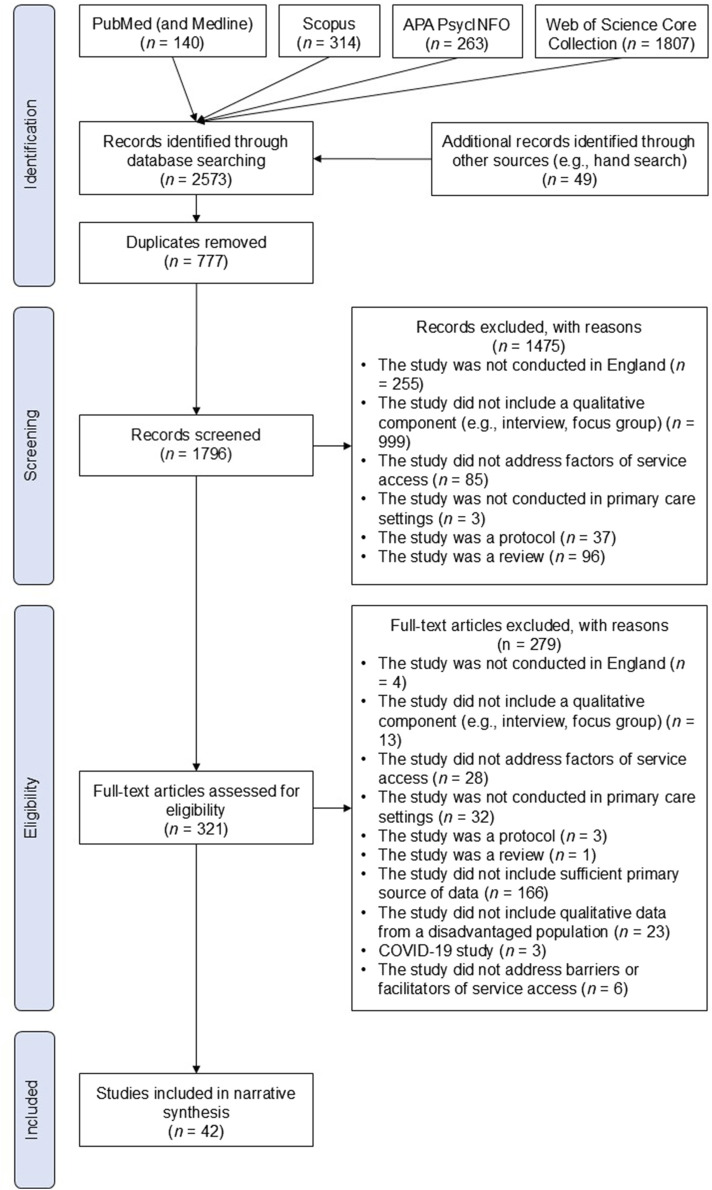
Preferred Reporting Items for Systematic Reviews and Meta-Analyses flow chart of the articles considered for the review (England, 2003–2024).

After removing duplicates, 1,796 unique records were screened. Based on title and abstract exclusions, 321 full texts were assessed. A total of 42 studies met the inclusion criteria and were included in the review (Figure 1).

### Study characteristics

Supplementary Table 4 presents the study characteristics, including sample characteristics, study setting, service focus, and data analysis approaches. The 42 included studies were published between 2005 and 2024 and were conducted across diverse locations in England, including the North of England (*n* = 8), Yorkshire (*n* = 7), the Northeast (*n* = 3), Northwest (*n* = 9), Midlands (*n* = 7), London (*n* = 7), Southeast (*n* = 1), and Southwest (*n* = 5), although some studies focused on specific cities and localities and a small number did not report a clearly defined location (*n* = 6). Sample sizes ranged from 8 to 90 participants, and the included populations represented overlapping forms of disadvantage, including ethnic minority and migrant communities (*n* = 18), people experiencing homelessness (*n* = 2), disabled people (*n* = 6), young people (*n* = 2), older adults (*n* = 3), people with long-term conditions (*n* = 16), people using substances (*n* = 9), asylum seekers and refugees (*n* = 2), and women with complex social and health-related vulnerabilities (*n* = 11). Participant characteristics frequently overlapped across studies, which reflected the intersecting social, economic, and health-related vulnerabilities represented in the review. The studies examined a broad range of statutory, voluntary-sector, and community-based services, including maternity care (*n* = 6), primary care (*n* = 2), mental health (*n* = 9), sexual health (*n* = 2), smoking cessation (*n* = 3), social prescribing (*n* = 2), welfare advice (*n* = 1), advocacy (*n* = 1), screening (*n* = 3), substance use (*n* = 2), migrant support (*n* = 7), homelessness support (*n* = 2), pharmacy services (*n* = 1), youth services (*n* = 2), and health promotion (*n* = 3). All studies used qualitative analytic approaches (*n* = 42), most commonly thematic analysis (*n* = 31) and framework-based analysis (*n* = 15). Other reported approaches included grounded theory-informed analysis (*n* = 2), constant comparative methods (*n* = 5), focused ethnography (*n* = 1), abductive thematic categorisation (*n* = 1), participatory evaluation (*n* = 1), and theory-informed approaches such as the Theoretical Domains Framework (*n* = 2), COM-B model (*n* = 1), Silences Framework (*n* = 1), and relational autonomy (*n* = 1).

### Quality assessment

The CASP quality appraisal showed that most studies demonstrated strong methodological quality, with 35 studies scoring 8 or above. Nearly all studies clearly stated aims (*n* = 41), used suitable methodologies (*n* = 42), and applied appropriate research designs (*n* = 41), recruitment strategies (*n* = 40), and data collection methods (*n* = 41). Most addressed ethical considerations (*n* = 39) and clearly conveyed their value (*n* = 41). The review team identified common limitations that included limited reflexivity around researcher-participant relationships (*n* = 33), unclear findings (*n* = 13), and inconsistent data analysis rigor (*n* = 10) (Supplementary Table 2).

### Synthesis of results

Four analytical themes were identified in the synthesis, each comprising of related barriers and facilitators represented through subthemes. The themes are presented in an order that reflects service engagement as a process that moves from factors shaping initial access, to the wider social and cultural contexts influencing engagement, to experiences within services, and finally to the emotional meanings and consequences associated with those encounters. Although analytically distinct, the themes were interconnected which reflected the complexity of service access and engagement.

### Pre-COVID and during/post-COVID differences

A descriptive comparison of subtheme representation suggested broad continuity rather than a marked shift between pre-COVID and during/post-COVID studies. All four themes were represented across both periods, which indicated that the core barriers and facilitators shaping access persisted over time. However, the clearest pattern was within the subtheme ‘The role of socioeconomic positioning in service access’, which appeared proportionally more often in during/post-COVID studies than in pre-COVID studies, with greater attention to communication, service responsiveness, and the practical demands of digitally mediated access. Community and locational enablers, cultural and linguistic influences, and discrimination, mistrust, and exclusion also remained prominent in the during/post-COVID studies. By contrast, some subthemes, particularly Engagement Motivated by Holistic Care and Lived Experience, were less visible in the during/post-COVID subset. Given the smaller number of during/post-COVID studies, these differences ought to be interpreted cautiously as these were represented by shifts in emphasis.

### Themes and subthemes identified

#### Theme 1: Structural and informational access: systems, pathways, and proximity

##### Structural and informational barriers to access

**FIGURE 2:**
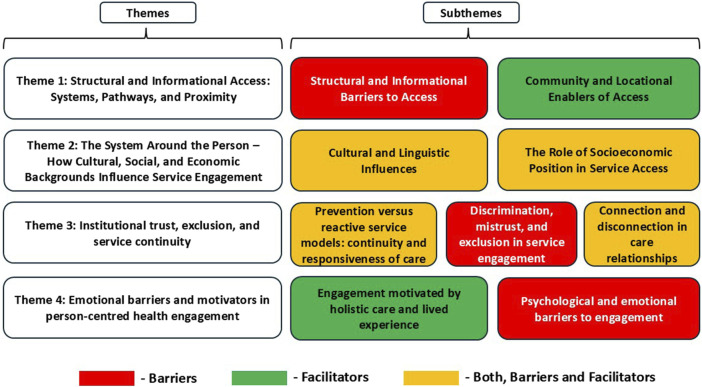
Visual Illustration of themes and subthemes (England, 2003–2024).

Structural and informational barriers were driven by limited awareness and unclear service pathways (Supplementary Table 5; Figure 2). Participants expressed uncertainty about what services were available [82], how to access them [83, 84] and what to expect [85]. Gaps in knowledge included basic understanding of service content [86] and referral processes [87]. These barriers were most explicit among racially minoritised groups and migrants, who reported that information was not communicated accessibly [84, 88, 89]. In some cases, structural requirements directly excluded families from entry into services. For example, one participant experiencing homelessness described how the requirement to provide a fixed address prevented registration altogether: “Any form you have you have to write your address, your postcode, your door number … When I fill any form without address it does not accept that … They did not give me appointment because I did not fill the form” (Samir) [90]. The lack of targeted outreach reduced confidence in accessing services [85]. This uncertainty is reflected in one account of an older Pakistani man who “…knew he should be getting some help, but he did not know how to go about it” and “…would not know where to go” (Case 3, Pakistani, male, aged 69, via interpreter, page 16) [84]. Medicalised language left parents unsure whether services were suitable [91, 92], while jargon [84, 92] and system complexity [30, 91] further undermined service coherence, especially in mental health [88, 93] and group-based interventions [83, 94]. Families described being left to navigate systems alone without guidance [84, 91, 95]. These structural deficits created both practical and psychological distance between families and services, reinforcing disengagement and deepening existing inequalities in access.

##### Community and locational enablers of access

Community and locational enablers of access were shaped by three interlinked factors: community-driven and peer-based support, trusted and familiar venues, and service proximity. In minoritised migrant communities [96], parents [97] and peers [98] were key sources of service information, with peer invitation and social familiarity encouraging engagement [99]. One participant described how information flowed through family and community networks, explaining, “We got all our information from the parents, aunty and uncle obviously, and we get all the information from them … mum knows everything that she needs to know” (No-MUR P7) [97]. This illustrates how informal interpersonal networks could act as important conduits of knowledge where formal communication pathways were limited. Trusted spaces, including community centres [99], cultural, and faith-settings [94], were seen as safe alternatives to intimidating mainstream services that were perceived as inaccessible [100]. Participants felt more at ease when services were delivered in places aligned with their daily routines [101, 102]. Service proximity was also critical for uptake [103], particularly where transport and travel time constrained access. As one participant noted, “It is not too far, I live next door to my GP surgery” (Majeeda, page 91) [104], which highlighted how physical closeness could make services feel more feasible and accessible.

#### Theme 2: The system around the person – the influence of cultural, social, and economic backgrounds on service engagement

##### Cultural and linguistic influences

Cultural and linguistic factors influenced service engagement through stigma, gender norms, and language barriers. Cultural stigma around mental health [105], help-seeking [97], and disclosure [106] led service users to conceal and minimise their health needs [106], particularly among communities where illness is interpreted as a spiritual punishment for weakness [86]. In some settings, health information itself was viewed as culturally threatening [107]. As a community leader explained, “Some of these sexual health teachings can be used to undermine our culture … We need to filter all the sexual health matters taught in communities to make sure that the community is not contaminated” (Jamu, male community leader, aged ≥35 years) [108]. Gendered cultural norms further constrained access, especially for women [87], who were expected to prioritise caregiving and defer health decisions [84, 93]. Language barriers compounded these challenges, impeding expression in sensitive health contexts. Participants described feeling dismissed [93], misunderstood [30], and dependent on family members for interpretation [89], which limited their autonomy. Despite these challenges, some accounts highlighted the positive impact of culturally and linguistically responsive services [109]. A service user noted in relation to faith-sensitive maternity support, that staff were “*brilliant*” and “accommodating of everything” (Interview #10, page 7) [110], when her birth plan included space for her husband to recite a prayer in the newborn baby’s ear. When practitioners demonstrated cultural competence [100, 110] and provided support in participants’ native languages [89], this helped to bridge gaps and foster engagement. However, these facilitators were less commonly experienced, pointing to persistent gaps in service equity for migrant and linguistically diverse communities.

##### The role of socioeconomic position in service access

Transport [98, 111], food, and fuel insecurity [112] restricted service use, especially for families with pregnant mothers [92, 110, 112] and young people [99]. A pregnant woman described that “I found it very difficult having to know that I’m pregnant, I am destitute, I have no money and no home, and then I’m going to have to pay this bill.” (Interview 2, page 5) [112]. Participants further reported missing appointments and avoiding engagement due to the cost of living [113] and the need to prioritise basic needs [30]. Digitalisation added to these barriers. The need for stable internet [113], phone credit [114], and digital literacy [94] excluded service users, with online booking systems and virtual communication often described as inaccessible [30]. Changes to GP phone lines [114] and the lack of adequate alternatives [92] created additional exclusion for low-income users. In this context, health services were perceived as inflexible and detached from lived realities. However, some participants described responsive forms of support, particularly where practical barriers were directly addressed. For example, one participant explained that patient transport services “take me where I have to go and pick me up and take me home as well” (Int4, female) [98]. Service users noted responsive service providers who offered in-person information and more direct communication [110], though efforts were found to be constrained by lack of funding [30, 99]. In the descriptive comparison by study period (Supplementary Table 3), this subtheme appeared to include relatively more reported facilitators in during/post-COVID studies than in pre-COVID studies, particularly among young people [99], homeless individuals [94], ethnic minorities [110], and those with substance use issues [113], although this pattern should be interpreted cautiously given the smaller number of during/post-COVID studies.

#### Theme 3: Institutional trust, exclusion, and service continuity

##### Prevention versus reactive service models: continuity and responsiveness of care

Service users’ experiences were shaped by rigid, crisis-led service models and fragmented pathways that disrupted continuity. Support was described as offered only at the point of breakdown [111], with preventative [99] and ongoing care consistently unavailable [82]. This reactive orientation is reflected in a service user’s account of seeking urgent help from primary care: “I went to the GP, I asked for emergency appointment because I have really emergency thing, but they said we cannot do that because we are crowded with the people in the queue…” (Tareq) [90]. Mental health [99] and substance use services [82, 113, 115], were marked by long wait lists and repeated assessments that rarely led to meaningful engagement. Users also encountered impersonal care [95], rotating staff [95, 106] and poor follow-up [30], with inadequately coordinated service transitions that were reliant on individual persistence [90, 95, 111]. These conditional systems left service users to fall through gaps, especially those from marginalised groups who reported feeling ignored [30, 113]. By contrast, services that offered continuity and relational support were experienced more positively. A Somali mother described how “the midwife come and see you and the health visitors come and see you, and then they keep on seeing you … guiding us,…” indicating how ongoing contact and repeated follow-up could create a sense of dependable support (Somali mother, 34 years). Therefore, services offering consistent, informal contact [113] and embedded advocacy were viewed as reliable and safe [91, 100]. Flexible support that responded to personal circumstances, rather than fixed thresholds, helped maintain engagement, even in crisis [102, 116]. Where trust was built through sustained relationships, care was perceived as more acceptable and effective.

##### Discrimination, mistrust, and exclusion in service engagement

Service engagement was also shaped by experiences of racial discrimination [106, 108], stereotyping [104, 105], and cultural invalidation [30, 110], specifically among racially minoritised individuals. Participants described being dismissed and disbelieved by health professionals, in maternity [110] and postnatal care [117], where assumptions based on ethnicity led to inadequate responses [112]. Staff was recorded to treat a service user “like I was thick,” speaking “loudly and slowly assuming I did not know English” and handling her “quite vicious[ly]” while trying to support infant feeding; she concluded that they were “ill-informed, racist, and rude” (Interview 14, page 4) [108]. Others reported being treated with suspicion [95] or facing overt prejudice [94], such as judgment attitudes [94] and inappropriate questioning [93]. An expectant mother was told by a midwife, “Why do not you go back where you came from?”, before criticising her pregnancy in relation to treatment costs (Survey respondent #28, page 9) [110]. Similarly, miscommunication and language barriers were frequently met with patronising behaviour rather than support [93], further compounding fear and mistrust [102]. These dynamics created a sense of exclusion, leading service users to avoid future engagement due to feeling unsafe and unwelcome [93, 101, 113]. Participants highlighted institutional exclusion through a lack of appropriate considerations, resulting in differential treatment across care settings [96, 104, 114, 117], from GPs to hospital wards, which undermined confidence in health systems.

##### Connection and disconnection in care relationships

Participants described meaningful connections formed through empathy [82] and informal interactions [105] with care providers. Such relationships developed when staff actively listened [84] and allowed space for service users to speak openly without fear of judgment [82]. These dynamics were valued in community and voluntary services [83, 102], where less hierarchical structures encouraged positive communication [118]. Link workers were described as “very friendly” and someone who were “there to just, generally, talk to” adding that “it was not as though she was like a worker … It was that good” (P4, female, age 55–59) [101]. Providers who built rapport [101], adapted to individual needs [117], and followed through on commitments were perceived as reliable [82]. In contrast, disconnection arose when professionals in statutory services seemed rushed or detached [30, 83]. As one participant explained, “they do not take time to listen to you” and interactions could leave people feeling “just like the next person” rather than recognised as individuals (Female F. FG1, page 5) [30]. Such experiences contributed to feeling misunderstood [30] and distant from providers [119].

#### Theme 4: Emotional barriers and motivators in person-centred health engagement

##### Engagement motivated by holistic care and lived experience

Services perceived as ethical and non-judgemental were seen to foster trust and emotional comfort, particularly in contexts where stigma was expected [82]. Participants valued care that extended beyond immediate medical needs [108], advocating for holistic models that addressed intersecting social, psychological, and practical challenges [100, 101]. The importance of this relational approach is reflected in a service user’s maternity support, where “the counselling was helpful, very, very helpful” because the midwife repeatedly encouraged her to “be strong” and to “focus on children” (KII 3, page 7) [100]. This was emphasised by ethnic minority community members, who called for integrated, culturally responsive services and expressed a desire to help shape care [86, 108]. Interactions prioritising empathy and relational continuity, such as with midwives [100] and link workers [101], contrasted with brief, impersonal clinical encounters [120]. Personal values, lived experiences of illness, and loss also shaped motivation were important in this context as a service user described “I had a very close friend who died of it, we were for many years close. So, and erm, I saw the whole process as such, I was with him throughout the period until he passed away… When you’ve seen someone close going through that process, then you understand …” [86], while others sought to protect future generations and to address past omissions [82, 86].

##### Psychological and emotional barriers to engagement

However, fear [112], shame [99], and perceived judgment [119] undermined service engagement across settings, especially where past experiences of neglect and stigma had eroded trust [95, 102]. Emotional overwhelm intensified during vulnerable moments including caregiving [86], pregnancy [121], and crisis [95], made it difficult to seek support when services lacked sensitivity. For example, attending A&E and being kept waiting “*2–3 h*” made service users feel “like you are being abandoned”, which “just worsen[ed] the situation” (KII 8, page 6) [100]. Environmental and interpersonal cues, such as feeling ignored [113] and reprimanded [95], discouraged continued contact, with formal health centres often viewed as unwelcoming compared to informal, peer-based settings [115]. Shame was linked to poverty, homelessness, substance use, and sexual activity; and contributed to self-silencing and service avoidance [82, 113, 122], as reflected in one participant’s statement: “The pain I’m going through I just cannot tell anyone about it coz I’m embarrassed” (M, aged 50 years, SC, page 532) [95]. Impersonal and culturally mismatched services added further emotional strain, particularly for migrant women [89]. Among youth, concerns around gossip and confidentiality breaches discouraged use of school-based services [99, 122].

## Discussion

### Access is a negotiated process

This co-produced qualitative review aimed to synthesise qualitative evidence on the perceived barriers and facilitators of accessing statutory and non-statutory health and social care services among disadvantaged communities in England. The thematic analysis found that disadvantaged communities experienced service access as difficult to initiate and sustain because services were fragmented, hard to understand, culturally and linguistically inaccessible, and poorly aligned with the material constraints of everyday life. Engagement was facilitated when support was local, trusted, relationally consistent, culturally responsive, and able to address practical and emotional needs alongside formal care. The overall findings are supported by international evidence and WHO guidance on community-centred and integrated care that shows access improves when services respond to the social and economic conditions shaping inequality and when local provision is organised in ways that reduce fragmentation [9–11]. The review also explored health equity [123] and intersectionality [124] perspectives, which placed engagement within wider structures of poverty, discrimination, exclusion, and unequal resource distribution [16–18, 26]. Consequently, access depended on whether services remained practically usable across successive encounters within the structural, relational, and emotional pressures of everyday life. The sections below, follow this argument by examining four cross-cutting mechanisms (Supplementary Table 5), navigation, trust, emotional and psychological safety, and service alignment, that connected the analytical themes across the findings. The cross-cutting mechanisms move beyond a descriptive account of barriers and facilitators and are used to demonstrate the relationships between the themes, and to show how disadvantage accumulated across the access pathway.

### Navigation as unequal labour

Accessing and navigating services depended on confidence, transport, language proficiency, and familiarity with institutional procedures. However, these resources were often unavailable among disadvantaged groups, especially where structural and intersecting forms of disadvantage already shaped everyday life. A Norwegian study involving Syrian refugees similarly confirmed that service users may formally have entitlement to care, but still face difficulties because navigating the system requires understanding how it works and dealing with unfamiliar institutions [125]. These demands in the review, shaped whether services were practically reachable. Navigation therefore emerged as an unevenly distributed burden, which highlighted how access depended on both service permeability [22, 24] and the practical and communicative capacities users needed to seek and reach care [19, 126]. Routine administrative requirements therefore became exclusionary, requiring repeated effort from users with the least capacity to absorb those costs. This interpretation is consistent with research from the USA [127] and Argentina [128] on treatment burden in healthcare, which shows that learning and compliance are often shifted onto users. Contrasting evidence highlighted that remote service can work better in rural settings with low-income adults, which may reflect access barriers rather than rejection of remote care itself [129].

Communication styles also added to this burden, in the current review. Unclear information, poorly explained pathways, and professionalised and medicalised language left users to interpret systems that were already difficult to approach. The negative effects of these challenges were previously confirmed whereby communication problems were found to be part of the burden itself that are not separate from administration [127]. Such issues arose from institutional fluency that could not be assumed among disadvantaged groups. This aligns with health equity research showing that engagement depends on unequal access to the material and social resources needed to use care [18, 26]. Community-linked and peer-based approaches reduced these difficulties by making services easier to find and enter through familiar settings and social relationships, consistent with evidence from the USA [47] and Zimbabwe [48] showing that community-based and outreach-oriented models can improve uptake and timeliness of care in underserved populations. Although previous literature indicated that service user navigation improved screening and reduced time to treatment initiation [130], our findings extend this evidence by indicating that navigation itself operated as a stratified demand, with the greatest burden falling on those least resourced to manage complexity and repeated effort.

### Trust as a precondition for engagement

Trust shaped whether services were entered and re-entered, with engagement tied to whether services were experienced as dependable. This suggests that trust was a key condition of access and played a more foundational role than in service models that place more emphasis on post-entry care [131]. Trust appeared to be already active before contact, especially where earlier encounters involved dismissal, stereotyping, cultural invalidation, and poor follow-up, thus suggesting that willingness to re-enter services was being shaped before access even occurred [22, 132]. This supports existing literature by showing that prior experiences of care can shape future candidacy before a new service encounter begins [22]. On the other hand, mistrust developed cumulatively through repeated encounters with fragmented pathways and experiences of dismissal and stereotyping. These patterns were most visible among racially minoritised participants. Such findings are line with previous evidence that showed marginalised patients’ experiences with restrictive healthcare systems and clinicians whose biased behaviour and lack of interest undermined meaningful care conversations [133]. Also, long waits and crisis-led thresholds left participants uncertain that help would follow through, further aligning with evidence that integrated care can improve user experience [50] and continuity [52]. However, trust was strengthened through everyday encounters in which service users experienced empathy and informal contact from providers. These experiences shaped whether support felt dependable and worth returning to, in line with evidence from Canada showing that culturally competent, co-designed services can build trust and improve uptake [42]. Previous evidence also highlighted that peer support and volunteer schemes can facilitate access by offering advocacy [53]. In the current review community and voluntary services were often experienced more positively, because their less hierarchical and more locally embedded forms of care helped service users feel that their needs and circumstances were understood, while also supporting repeated contact.

### Emotional and psychological safety as foundational

Emotional and psychological safety shaped whether support felt possible to disclose during service engagement. Fear, shame, anticipated judgement, and emotional overwhelm appeared to restrict engagement across a range of settings, particularly where services were experienced as insensitive to vulnerability. In these circumstances, emotional safety set the terms on which support could be approached and sustained, as research in similarly marginalised contexts has likewise shown [134]. Such user responses were closely tied to prior interactions and service environments rather than to individual reluctance, with formal health settings often experienced as more dismissive and judgemental than informal and peer-based environments. However, evidence from marginalised populations shows that formal healthcare settings can also become safer and more supportive when providers use destigmatising practices [135] and receive training that increases empathy and reduces prejudice toward underserved patients [136]. The findings also demonstrated service users experiencing shame, which was linked to homelessness, substance use, sexual activity, and other stigmatised experiences, while concerns about gossip and reputational harm were particularly salient in youth and school-based settings. Shame and fear therefore appeared as socially patterned responses, shaped by what services communicated about who could speak safely and on what terms. Previous evidence supports these observations by indicating that shame acts as a barrier to speaking out and disclosing problems to professionals because of expectations of judgment [137].

In terms of how vulnerability was conceptualised, emotional strain also intensified during periods of heightened care and dependency, such as pregnancy and caregiving, which supports wider research showing that vulnerability is shaped by individual need and by services’ response to acute dependence [134]. In the current review, where that sensitivity was absent, service contact became harder to initiate and maintain even when provision was available. By contrast, opportunities to speak openly without fear of dismissal, within care was experienced as ethical and non-judgemental, and helped create the psychological safety needed for continued engagement. This suggests that access was strengthened through care that reduced emotional threat and made disclosure feel safer, which is in keeping with international evidence that culturally responsive and person-centred forms of care can improve access and resilience and that relational models can support health through emotionally supportive engagement [13, 53].

### Alignment versus misalignment in service engagement

Continued engagement was more likely where services aligned with cultural expectations and socioeconomic realities. This suggests that access depended on service availability and on the fit between service organisation and users’ ability to seek, reach, and engage with care [19, 126]. Across the findings, support became easier to use when it was delivered through familiar settings, communicated in culturally and linguistically intelligible ways, and organised around the practical constraints of childcare, work, and financial strain. However, where that fit was absent, availability did not translate into use, because services assumed economic flexibility and stability [19, 138], in line with evidence from the USA that demonstrates ordinary service arrangements can become unusable when they are designed around economic assumptions that do not mirror the lives of many users [139].

On the other hand, misalignment led to disengagement even where services were formally available. The need to prioritise basic needs made support harder to reach, subsequently reflecting wider evidence that service users’ financial situations and fragmented service geographies can constrain access for disadvantaged communities [27, 37, 39]. Digital-first systems and service designs that assumed stable internet and phone access also made engagement harder to initiate and sustain, supported by evidence that digital tools can reproduce inequalities when differences in physical access and digital access are not addressed [57, 59]. In this sense, some services appeared misaligned practically and socially, and where services failed to recognise culturally shaped understandings of help-seeking, support became harder to approach and easier to withdraw from. UK-based evidence similarly demonstrated that cultural mismatches can reduce engagement through bias and limited cultural competence [30, 31], while evidence from South Korea [41] and Canada [42] showed that culturally competent, co-designed services can improve uptake among disadvantaged communities.

### Implications of the pre-COVID and during/post-COVID comparison

The comparison suggests that the pandemic did not fundamentally alter the overall structure of access inequities, but it appeared to have exposed and, in some instances, might have intensified pre-existing disadvantages. The during/post-COVID studies placed greater emphasis on socioeconomic positioning, particularly the practical constraints associated with service responsiveness, remote contact, and digital access. This suggests that digital exclusion may have amplified inequalities for disadvantaged groups, especially where access depended on stable internet, phone credit, digital literacy, and confidence in dealing with altered systems. Also, the continued prominence of community and locational enablers indicates that familiar, trusted, and locally embedded forms of support remained important routes into care. Thus, the post-pandemic learning point is to ensure that services are flexible, multimodal, culturally and linguistically responsive, and organised in ways that reduce the unequal labour of accessing support.

### Theoretical implications

The findings extend Levesque et al.’s framework [19] by highlighting how access inequities accumulated across the care pathway in disadvantaged contexts. Early difficulties in perceiving, seeking, and reaching care often constrained later possibilities for obtaining and engaging with support [19]. Subsequently, service access operated as a cumulative process in which limited institutional familiarity and material insecurity progressively narrowed opportunities for action. The implication is a move away from treating barriers as discrete interruptions in access and towards understanding how systems can organise disadvantage over time. In relation to Candidacy Theory [22], access was shaped by the practical and social demands involved in becoming intelligible to services. Service permeability and judgements of need were points at which disadvantage could improve or deteriorate. The findings add specificity by showing that access depended on whether service users could make claims in forms that institutions recognised as credible, possessed the social and communicative resources expected by services, and felt able to re-enter after earlier encounters. Trust, shame, and emotional safety therefore formed part of the production of eligibility itself. The two frameworks are connected by the question of fit, which operated as a selection mechanism embedded in ordinary service design. Formal provision was converted into unequal access when routine service expectations presupposed temporal flexibility, institutional confidence, communicative fluency, and material stability more typical of advantaged social positions. This helps explain how service-user fit and institutional permeability can turn routine arrangements into mechanisms of exclusion.

The Intersectionality [124] perspective demonstrated why the above restrictions are unequally distributed. In the current review, access is characterised by institutional production of exclusion, where disadvantage is generated through service rules, professional judgement, communication norms, and eligibility practices that organise access unevenly. This means that such concepts are better understood as social relations that structure how services are encountered, whose claims are more readily recognised, and which forms of disadvantage are more easily converted into delay or exclusion. The findings also supported Health equity [123] stance on the institutional organisation of unequal access by indicating that inequity was routed in the routine design of services, which shaped whose needs were more easily articulated, were more likely to endure, and where disengagement became more likely. The implication is that access inequality should be understood as a property of service systems as well as a pattern in user outcomes, with disadvantage reproduced where ordinary service expectations are aligned with more advantaged social positions.

### Implications for policy and practice

The findings suggest that improving access requires commissioning and service models that are explicitly organised around equity, rather than assuming that availability alone will produce uptake. In practice, this means using flexible, outcomes-based commissioning arrangements that support hybrid delivery across outreach, in-person, and digital routes, and that allow providers to adapt how support is offered in response to local barriers to access. Within ICSs, co-production should be embedded as a routine feature of governance and commissioning, with funded and ongoing involvement of service users and VCFSE partners in decision-making, and with clear accountability for demonstrating how lived experience has shaped service design, delivery, and review. Access is also likely to depend on culturally and linguistically responsive provision, protected non-digital routes into care, and relational continuity for people with complex or intersecting needs. In this context, relational continuity should be treated as a measurable quality outcome, assessed through consistency of contact, follow-up, reduced need to repeat personal histories, and service users’ experiences of trust and emotional safety.

### Strength and limitations

Co-produced with contributors from the VCFSE sector, academia, and the public, the review was grounded in the lived realities of marginalised communities. Its focus on qualitative self-reported accounts of service access enabled an in-depth and multi-dimensional analysis of how structural, relational, cultural, and emotional conditions shaped engagement, which strengthened the review’s health equity perspective. Some limitations should also be noted. The review included only English-language publications and studies conducted in England, which reflected its focus on service access within the English health and social care context; however, the volume of relevant literature from England made this a worthwhile and policy-relevant undertaking. On the other hand, this may have excluded potentially relevant insights from similar disadvantaged settings elsewhere. Grey literature was excluded that may have contained additional qualitative evidence relevant to community-based service access.

### Future studies

Future research should build on the gaps in representation within the current synthesis. Greater attention is needed to voices where forms of disadvantage intersect in ways that remain underexplored, including combinations of ethnicity, gender, disability, migration status, sexuality, and poverty. More evidence is also needed from underrepresented service contexts and community-based models, to clarify how peer-led and community-led provision operates in practice. Given the exclusion of grey literature and the restriction to English-language studies conducted in England, future research would benefit from drawing on a wider range of community-based evidence and service settings. Longitudinal and co-produced research would be valuable in showing how access is shaped across repeated encounters rather than at single points of contact.

### Conclusion

In conclusion, this review highlights that service access depends not only on whether services exist, but on how they are experienced and aligned with people’s lived realities. For disadvantaged service users, unclear pathways, cultural mismatch, financial strain, and low institutional trust limit their ability to engage with care. Improving access therefore requires service design that responds to the social, material, and emotional realities of people’s lives. This includes working with service users to shape care that is relevant and responsive. Co-production can support this, but only if it addresses structural barriers and supports equal participation. For planners and commissioners, the challenge is to move beyond provision alone and toward services that are approachable, consistent, and meaningfully shaped with the communities they aim to support.
