# Supplementary material for: Perceived barriers and facilitators of accessing statutory and non-statutory services, in disadvantaged communities, in England: a co-produced qualitative review
Source: Public Health Rev. 2026 May 28;47:1608969. doi: 10.3389/phrs.2026.1608969 (PMC13377981; doi:10.3389/phrs.2026.1608969)
Supplement: Supplementary file 1 [file Supplementaryfile1.docx]

Supplementary Material 1: Search term formats used during the literature search

Web of Science: (Database limits: 2003-2024)

(TS=access OR TS=‘service access’ OR TS=barrier OR TS=‘barrier* to access’ OR TS=‘access barrier’ OR TS=‘access challenge’ OR TS=facilitator OR TS=‘access to support’ OR TS=‘use of service*’ OR TS=‘usage of service*’ OR TS=‘using* service*’ OR TS=‘service use*’ OR TS=‘service usag*’ OR TS=‘utili* of service*’ OR TS=‘service utili*’) AND (TS=‘community health service’ OR TS=‘social care service’ OR TS=‘community-based clinic’ OR TS=‘local health centre’ OR TS=‘community health program’ OR TS=‘neighbourhood health centre’ OR TS=‘community healthcare’ OR TS=‘community-level health service’ OR TS=‘non-hospital healthcare service’ OR TS=‘primary care’ OR TS=‘community healthcare centre’ OR TS=‘community-based statutory service’ OR TS=‘non-statutory service*’ OR TS=‘social prescr*’ OR TS=’community-hub’ OR TS=GP OR TS=ICB*) AND (TS=england* OR TS=united kingdom* OR TS=great britain* OR TS=britain* OR TS=bedfordshire* OR TS=luton* OR TS=milton keynes* OR TS=cambridgeshire* OR TS=peterborough* OR TS=hertfordshire* OR TS=west essex* OR TS=mid essex* OR TS=south essex* OR TS=norfolk* OR TS=waveney* OR TS=suffolk* OR TS=north east essex* OR TS=north central london* OR TS=north east london* OR TS=north west london* OR TS=south east london* OR TS=south west london* OR TS=birmingham* OR TS=solihull* OR TS=black country* OR TS=coventry* OR TS=warwickshire* OR TS=derby* OR TS=derbyshire* OR TS=herefordshire* OR TS=worcestershire* OR TS=leicester* OR TS=leicestershire* OR TS=rutland* OR TS=lincolnshire* OR TS=northamptonshire* OR TS=nottingham* OR TS=nottinghamshire* OR TS=shropshire* OR TS=telford* OR TS=wrekin* OR TS=staffordshire* OR TS=stoke-on-trent* OR TS=humber* OR TS=north yorkshire* OR TS=north east* OR TS=north cumbria* OR TS=south yorkshire* OR TS=west yorkshire* OR TS=cheshire* OR TS=merseyside* OR TS=greater manchester* OR TS=lancashire* OR TS=south cumbria* OR TS=buckinghamshire* OR TS=oxfordshire* OR TS=berkshire west* OR TS=frimley* OR TS=hampshire* OR TS=isle of wight* OR TS=kent* OR TS=medway* OR TS=surrey heartlands* OR TS=sussex* OR TS=bath* OR TS=north east somerset* OR TS=swindon* OR TS=wiltshire* OR TS=bristol* OR TS=north somerset* OR TS=south gloucestershire* OR TS=cornwall* OR TS=the isles of scilly* OR TS=devon* OR TS=dorset* OR TS=gloucestershire* OR TS=somerset*) AND (TS=deprivation OR TS=deprived OR TS=marginalised OR TS=under-represented OR TS=under-served OR TS=poverty OR TS=disadvantage* OR TS=impoverish* OR TS=inequ* OR TS=disp*)

Scopus: (Database limits: 2003-2024)

(TITLE-ABS-KEY(access) OR TITLE-ABS-KEY(‘service access’) OR TITLE-ABS-KEY(barrier) OR TITLE-ABS-KEY(‘barrier* to access’) OR TITLE-ABS-KEY(‘access barrier’) OR TITLE-ABS-KEY(‘access challenge’) OR TITLE-ABS-KEY(facilitator) OR TITLE-ABS-KEY(‘access to support’) OR TITLE-ABS-KEY(usage of service*) OR TITLE-ABS-KEY(using* service*) OR TITLE-ABS-KEY(service use*) OR TITLE-ABS-KEY(service usag*) OR TITLE-ABS-KEY(utili* of service*) OR TITLE-ABS-KEY(service utili*) AND TITLE-ABS-KEY(‘community health service’) OR TITLE-ABS-KEY(‘social care service’) OR TITLE-ABS-KEY(‘community-based clinic’) OR TITLE-ABS-KEY(‘local health centre’) OR TITLE-ABS-KEY(‘community health program’) OR TITLE-ABS-KEY(‘neighbourhood health centre’) OR TITLE-ABS-KEY(‘community healthcare’) OR TITLE-ABS-KEY(‘community-level health service’) OR TITLE-ABS-KEY(‘non-hospital healthcare service’) OR TITLE-ABS-KEY(‘primary care’) OR TITLE-ABS-KEY(‘community healthcare centre’) OR TITLE-ABS-KEY(‘community-based statutory service’) OR TITLE-ABS-KEY(‘non-statutory service*’) OR TITLE-ABS-KEY(‘social prescr*’) OR TITLE-ABS-KEY(’community-hub’) OR TITLE-ABS-KEY(GP) OR TITLE-ABS-KEY(ICB*) AND TITLE-ABS-KEY(england*) OR TITLE-ABS-KEY(united kingdom*) OR TITLE-ABS-KEY(great britain*) OR TITLE-ABS-KEY(britain*) OR TITLE-ABS-KEY(bedfordshire*) OR TITLE-ABS-KEY(luton*) OR TITLE-ABS-KEY(milton keynes*) OR TITLE-ABS-KEY(cambridgeshire*) OR TITLE-ABS-KEY(peterborough*) OR TITLE-ABS-KEY(hertfordshire*) OR TITLE-ABS-KEY(west essex*) OR TITLE-ABS-KEY(mid essex*) OR TITLE-ABS-KEY(south essex*) OR TITLE-ABS-KEY(norfolk*) OR TITLE-ABS-KEY(waveney*) OR TITLE-ABS-KEY(suffolk*) OR TITLE-ABS-KEY(north east essex*) OR TITLE-ABS-KEY(north central london*) OR TITLE-ABS-KEY(north east london*) OR TITLE-ABS-KEY(north west london*) AND TITLE-ABS-KEY(south east london*) OR TITLE-ABS-KEY(south west london*) OR TITLE-ABS-KEY(birmingham*) OR TITLE-ABS-KEY(solihull*) OR TITLE-ABS-KEY(black country*) OR TITLE-ABS-KEY(coventry*) OR TITLE-ABS-KEY(warwickshire*) OR TITLE-ABS-KEY(rutland*) OR TITLE-ABS-KEY(lincolnshire*) OR TITLE-ABS-KEY(northamptonshire*) OR TITLE-ABS-KEY(nottingham*) OR TITLE-ABS-KEY(nottinghamshire*) OR TITLE-ABS-KEY(shropshire*) OR TITLE-ABS-KEY(telford*) OR TITLE-ABS-KEY(wrekin*) OR TITLE-ABS-KEY(staffordshire*) OR TITLE-ABS-KEY(stoke-on-trent*) OR TITLE-ABS-KEY(humber*) OR TITLE-ABS-KEY(north yorkshire*) OR TITLE-ABS-KEY(north east*) OR TITLE-ABS-KEY(north cumbria*) OR TITLE-ABS-KEY(south yorkshire*) OR TITLE-ABS-KEY(west yorkshire*) OR TITLE-ABS-KEY(cheshire*) OR TITLE-ABS-KEY(merseyside*) OR TITLE-ABS-KEY(greater manchester*) OR TITLE-ABS-KEY(lancashire*) OR TITLE-ABS-KEY(south cumbria*) OR TITLE-ABS-KEY(buckinghamshire*) OR TITLE-ABS-KEY(oxfordshire*) OR TITLE-ABS-KEY(berkshire west*) OR TITLE-ABS-KEY(frimley*) OR TITLE-ABS-KEY(hampshire*) OR TITLE-ABS-KEY(isle of wight*) OR TITLE-ABS-KEY(kent*) OR TITLE-ABS-KEY(medway*) OR TITLE-ABS-KEY(surrey heartlands*) OR TITLE-ABS-KEY(sussex*) OR TITLE-ABS-KEY(bath*) OR TITLE-ABS-KEY(north east somerset*) OR TITLE-ABS-KEY(swindon*) OR TITLE-ABS-KEY(wiltshire*) OR TITLE-ABS-KEY(bristol*) OR TITLE-ABS-KEY(north somerset*) OR TITLE-ABS-KEY(south gloucestershire*) OR TITLE-ABS-KEY(cornwall*) OR TITLE-ABS-KEY(the isles of scilly*) OR TITLE-ABS-KEY(devon*) OR TITLE-ABS-KEY(dorset*) OR TITLE-ABS-KEY(gloucestershire*) OR TITLE-ABS-KEY(somerset*) AND TITLE-ABS-KEY(deprivation) OR TITLE-ABS-KEY(deprived) OR TITLE-ABS-KEY(marginalised) OR TITLE-ABS-KEY(under-represented) OR TITLE-ABS-KEY(under-served) OR TITLE-ABS-KEY(poverty) OR TITLE-ABS-KEY(disadvantage*) OR TITLE-ABS-KEY(impoverish*) OR TITLE-ABS-KEY(inequ*) OR TITLE-ABS-KEY(disp*)) AND PUBYEAR > 2002 AND PUBYEAR < 2024

APA PsycInfo: (Database limits: 2003-2024)

(access OR ‘service access’ OR barrier OR ‘barrier* to access’ OR ‘access barrier’ OR ‘access challenge’ OR facilitator OR ‘access to support’ OR 'use of service*' OR 'usage of service*' OR 'using* service*' OR 'service use*' OR 'service usag*' OR 'utili* of service*' OR 'service utili*') AND (‘community health service’ OR ‘social care service’ OR ‘community-based clinic’ OR ‘local health centre’ OR ‘community health program’ OR ‘neighbourhood health centre’ OR ‘community healthcare’ OR ‘community-level health service’ OR ‘non-hospital healthcare service’ OR ‘primary care’ OR ‘community healthcare centre’ OR ‘community-based statutory service’ OR ‘non-statutory service*’ OR ‘social prescr*’ OR ’community-hub’ OR GP OR ICB*) AND (england* OR united kingdom* OR great britain* OR britain* OR bedfordshire* OR luton* OR milton keynes* OR cambridgeshire* OR peterborough* OR hertfordshire* OR west essex* OR mid essex* OR south essex* OR norfolk* OR waveney* OR suffolk* OR north east essex* OR north central london* OR north east london* OR north west london* OR south east london* OR south west london* OR birmingham* OR solihull* OR black country* OR coventry* OR warwickshire* OR derby* OR derbyshire* OR herefordshire* OR worcestershire* OR leicester* OR leicestershire* OR rutland* OR lincolnshire* OR northamptonshire* OR nottingham* OR nottinghamshire* OR shropshire* OR telford* OR wrekin* OR staffordshire* OR stoke-on-trent* OR humber* OR north yorkshire* OR north east* OR north cumbria* OR south yorkshire* OR west yorkshire* OR cheshire* OR merseyside* OR greater manchester* OR lancashire* OR south cumbria* OR buckinghamshire* OR oxfordshire* OR berkshire west* OR frimley* OR hampshire* OR isle of wight* OR kent* OR medway* OR surrey heartlands* OR sussex* OR bath* OR north east somerset* OR swindon* OR wiltshire* OR bristol* OR north somerset* OR south gloucestershire* OR cornwall* OR the isles of scilly* OR devon* OR dorset* OR gloucestershire* OR somerset*) AND (deprivation OR deprived OR marginalised OR under-represented OR under-served OR poverty OR disadvantage* OR impoverish* OR inequ* OR disp*)

PubMed and Medline: (Database limits: 2003-2024)

**access[Title/Abstract] OR ‘service access’[Title/Abstract] OR barrier[Title/Abstract] OR ‘barrier* to access’[Title/Abstract] OR ‘access barrier’[Title/Abstract] OR ‘access challenge’[Title/Abstract] OR facilitator[Title/Abstract] OR ‘access to support’[Title/Abstract]** OR ‘usage of service*’**[Title/Abstract]** OR ‘using* service*’**[Title/Abstract]** OR ‘service use*’**[Title/Abstract]**  OR service usage**[Title/Abstract]** OR ‘utili*ation of service*’**[Title/Abstract]** OR ‘service utili*ation’**[Title/Abstract] AND (‘community health service’[Title/Abstract] OR ‘social care service’[Title/Abstract] OR ‘community-based clinic’[Title/Abstract] OR ‘local health centre’[Title/Abstract] OR ‘community health program’[Title/Abstract] OR ‘neighbourhood health centre’[Title/Abstract] OR ‘community healthcare’[Title/Abstract] OR ‘community-level health service’[Title/Abstract] OR ‘non-hospital healthcare service’[Title/Abstract] OR ‘primary care’[Title/Abstract] OR ‘community healthcare centre’[Title/Abstract] OR ‘community-based statutory service’[Title/Abstract] OR ‘non-statutory service*’[Title/Abstract] OR ‘social prescr*’[Title/Abstract] OR ’community-hub’[Title/Abstract] OR GP[Title/Abstract] OR ICB*[Title/Abstract])) AND (england*[Title/Abstract] OR united kingdom*[Title/Abstract] OR great britain*[Title/Abstract] OR britain*[Title/Abstract] OR bedfordshire*[Title/Abstract] OR luton*[Title/Abstract] OR milton keynes*[Title/Abstract] OR cambridgeshire*[Title/Abstract] OR peterborough*[Title/Abstract] OR hertfordshire*[Title/Abstract] OR west essex*[Title/Abstract] OR mid essex*[Title/Abstract] OR south essex*[Title/Abstract] OR norfolk*[Title/Abstract] OR waveney*[Title/Abstract] OR suffolk*[Title/Abstract] OR north east essex*[Title/Abstract] OR north central london*[Title/Abstract] OR north east london*[Title/Abstract] OR north west london*[Title/Abstract] OR south east london*[Title/Abstract] OR south west london*[Title/Abstract] OR birmingham*[Title/Abstract] OR solihull*[Title/Abstract] OR black country*[Title/Abstract] OR coventry*[Title/Abstract] OR warwickshire*[Title/Abstract] OR derby*[Title/Abstract] OR derbyshire*[Title/Abstract] OR herefordshire*[Title/Abstract] OR worcestershire*[Title/Abstract] OR leicester*[Title/Abstract] OR leicestershire*[Title/Abstract] OR rutland*[Title/Abstract] OR lincolnshire*[Title/Abstract] OR northamptonshire*[Title/Abstract] OR nottingham*[Title/Abstract] OR nottinghamshire*[Title/Abstract] OR shropshire*[Title/Abstract] OR telford*[Title/Abstract] OR wrekin*[Title/Abstract] OR staffordshire*[Title/Abstract] OR stoke-on-trent*[Title/Abstract] OR humber*[Title/Abstract] OR north yorkshire*[Title/Abstract] OR north east*[Title/Abstract] OR north cumbria*[Title/Abstract] OR south yorkshire*[Title/Abstract] OR west yorkshire*[Title/Abstract] OR cheshire*[Title/Abstract] OR merseyside*[Title/Abstract] OR greater manchester*[Title/Abstract] OR lancashire*[Title/Abstract] OR south cumbria*[Title/Abstract] OR buckinghamshire*[Title/Abstract] OR oxfordshire*[Title/Abstract] OR berkshire west*[Title/Abstract] OR frimley*[Title/Abstract] OR hampshire*[Title/Abstract] OR isle of wight*[Title/Abstract] OR kent*[Title/Abstract] OR medway*[Title/Abstract] OR surrey heartlands*[Title/Abstract] OR sussex*[Title/Abstract] OR bath*[Title/Abstract] OR north east somerset*[Title/Abstract] OR swindon*[Title/Abstract] OR wiltshire*[Title/Abstract] OR bristol*[Title/Abstract] OR north somerset*[Title/Abstract] OR south gloucestershire*[Title/Abstract] OR cornwall*[Title/Abstract] OR the isles of scilly*[Title/Abstract] OR devon*[Title/Abstract] OR dorset*[Title/Abstract] OR gloucestershire*[Title/Abstract] OR somerset*[Title/Abstract])) AND (deprivation[Title/Abstract] OR deprived[Title/Abstract] OR marginalised[Title/Abstract] OR under-represented[Title/Abstract] OR under-served[Title/Abstract] OR poverty[Title/Abstract] OR disadvantage*[Title/Abstract] OR impoverish*[Title/Abstract] OR** inequ***Title/Abstract]** OR dispar***[Title/Abstract]** OR dispro***[Title/Abstract]**
