# Supplementary material for: Perceived barriers and facilitators of accessing statutory and non-statutory services, in disadvantaged communities, in England: a co-produced qualitative review
Source: Public Health Rev. 2026 May 28;47:1608969. doi: 10.3389/phrs.2026.1608969 (PMC13377981; doi:10.3389/phrs.2026.1608969)
Supplement: Supplementary file 2 [file Supplementaryfile5.docx]

**Table 5. Themes and subthemes with key barriers and facilitators, illustrative processes, and cross-cutting mechanisms described (England, 2003–2024)**

| **Themes** | **Subthemes** | **Key Barriers** | **Key Facilitators** | **Illustrative processes** | **Cross-cutting mechanisms** |
| --- | --- | --- | --- | --- | --- |
| Theme 1: Structural and Informational Access: Systems, Pathways, and Proximity | Structural and Informational Barriers to Access (30,82,83,85–95,97,98,100,104,110–112,116,121) | Limited awareness; unclear pathways; inaccessible information; jargon; no guidance | N/A | Unclear communication left families unsure how services worked | Navigation, Service alignment |
|  | Community and Locational Enablers of Access (82,85,87,90,94–106,109,111,113,115,118,122) | N/A | Peer support; trusted venues; local delivery | Familiar places and peer invitation reduced intimidation and travel burden |  |
| Theme 2: The System Around the Person – How Cultural, Social, and Economic Backgrounds Influence Service Engagement | Cultural and Linguistic Influences (30,86–89,93,94,97,98,100–102,105–110,114,116,118–122) | Stigma; gender norms; language barriers; cultural misunderstanding | Culturally competent care; native-language support | Cultural stigma constrained help-seeking; culturally matched communication improved engagement | Navigation, Service alignment |
|  | The Role of Socioeconomic Positioning in Service Access (30,85,92,94,95,98–100,110–114,119) | Poverty-related costs; digital exclusion; inflexible systems | Direct communication; practical flexibility | Basic-needs pressures and digital requirements displaced service use |  |
| Theme 3: Institutional Trust, Exclusion, and Service Continuity | Prevention versus Reactive Service Models: Continuity and Responsiveness of Care (30,82,90,91,95,99,100,102,106,111,113–116,122) | Crisis-led care; long waits; poor follow-up; fragmented care | Preventive care; consistent contact; advocacy; flexibility | Help arrived late in fragmented systems; sustained support improved continuity | Navigation, Trust, Emotional and psychological safety, Service alignment |
|  | Discrimination, Mistrust, and Exclusion in Service Engagement (30,85,89,93–95,97–99,101,102,104–108,110–114,117–122) | Racism; stereotyping; invalidation; mistrust | N/A | Disbelief and differential treatment made services feel unsafe |  |
|  | Connection and Disconnection in Care Relationships (30,82–86,92,93,97,98,101,102,104,105,110,113,114,116–119) | Detached staff; poor communication | Empathy; listening; rapport | Listening and follow-through built trust; rushed encounters created distance |  |
| Theme 4: Emotional Barriers and Motivators in Person-Centred Health Engagement | Engagement Motivated by Holistic Care and Lived Experience (82,86,100,101,108,120,121) | N/A | Holistic non-judgemental care; continuity; user involvement | Broader, person-centred care aligned with lived realities and strengthened motivation | Trust, Emotional and psychological safety, Service alignment |
|  | Psychological and Emotional Barriers (82,85,86,89,91,92,95,99–102,112–115,117,120–122) | Fear; shame; judgment; confidentiality concerns | N/A | Anticipated criticism demotivated disclosure and re-entering |  |
