# Supplementary material for: Perceived barriers and facilitators of accessing statutory and non-statutory services, in disadvantaged communities, in England: a co-produced qualitative review
Source: Public Health Rev. 2026 May 28;47:1608969. doi: 10.3389/phrs.2026.1608969 (PMC13377981; doi:10.3389/phrs.2026.1608969)
Supplement: Supplementary file 3 [file Supplementaryfile3.docx]

**Table 3. Themes and subthemes represented in each study (England, 2003–2024)**

|  | | Themes and Subthemes, with changes marked in subtheme representations Pre- vs. During and post-COVID-19 for each subtheme | | | | | | | | |
| --- | --- | --- | --- | --- | --- | --- | --- | --- | --- | --- |
|  |  | Theme 1 Structural and Informational Access: Systems, Pathways, and Proximity | | Theme 2 The System Around the Person – How Cultural, Social, and Economic Backgrounds Influence Service Engagement | | Theme 3 Institutional Trust, Exclusion, and Service Continuity | | | Theme 4 Emotional Barriers and Motivators in Person-Centred Health Engagement | |
| **Authors and Year - Data collected Pre-COVID-19** | **Disadvantaged population** | **Structural and Informational Barriers to Access** | **Community and Locational Enablers of Access** | **Cultural and Linguistic Influences** | **The Role of Socioeconomic Positioning in Service Access** | **Prevention versus Reactive Service Models: Continuity and Responsiveness of care** | **Discrimination, Mistrust, and Exclusion in Service Engagement** | **Connection and Disconnection in Care Relationships** | **Engagement Motivated by Holistic Care and Lived Experience** | **Psychological and Emotional Barriers** |
| Jayaweera et al., 2005 **(104)** | Bangladeshi women | ✓ | ✓ |  |  |  | ✓ | ✓ |  |  |
| Higginbottom, 2006 **(105)** | African Caribbean with hypertension |  | ✓ | ✓ |  |  | ✓ | ✓ |  |  |
| Roddy et al., 2006 **(85)** | Socioeconomically deprived | ✓ | ✓ |  | ✓ |  | ✓ | ✓ |  | ✓ |
| Moffatt et al., 2009 **(84)** | South Asian |  |  |  |  |  |  | ✓ |  |  |
| Coles et al., 2010 **(119)** | Socioeconomically deprived |  |  | ✓ | ✓ |  | ✓ | ✓ |  |  |
| Ingram et al., 2010 **(122)** | Socioeconomically deprived |  | ✓ | ✓ |  | ✓ | ✓ |  |  | ✓ |
| Baxter et al., 2011 **(91)** | Disabled adults | ✓ |  |  |  | ✓ |  |  | ✓ |  |
| Leite et al., 2011 **(93)** | Ethnic minority, socioeconomically deprived , and chronic illness | ✓ |  | ✓ |  |  | ✓ | ✓ |  |  |
| Williams et al., 2012 **(120)** | Socioeconomically deprived |  |  | ✓ |  |  | ✓ |  | ✓ |  |
| Newbigging et al., 2013 **(96)** | African and Caribbean |  | ✓ |  |  |  |  |  |  |  |
| Ochieng, B. M. N. 2013 **(88)** | Black African migrant | ✓ |  | ✓ |  |  |  |  |  |  |
| Blickem et al., 2013 **(103)** | Long-term health conditions and socioeconomically deprived |  | ✓ |  |  |  |  |  |  |  |
| Haddrill et al., 2014 **(121)** | Ethnic minority and socioeconomically deprived | ✓ |  | ✓ |  |  | ✓ |  | ✓ | ✓ |
| Bains et al., 2015 **(115)** | Socioeconomically deprived |  | ✓ |  |  | ✓ |  |  |  | ✓ |
| Mastrocola et al., 2015 **(114)** | Street-based prostitution, long-term physical and mental health conditions, and socioeconomically deprived |  |  | ✓ | ✓ | ✓ | ✓ | ✓ |  | ✓ |
| Memon et al., 2016 **(30)** | Black and other minority ethnic | ✓ |  | ✓ | ✓ | ✓ | ✓ | ✓ |  |  |
| Islam, M. P. 2016 **(92)** | Socioeconomically deprived | ✓ |  |  | ✓ |  |  | ✓ | ✓ | ✓ |
| Nyashanu et al., 2016 **(108)** | Black sub-Saharan African |  | ✓ | ✓ |  |  |  |  |  |  |
| Dharni et al., 2017 **(86)** | Socioeconomically deprived | ✓ |  | ✓ |  |  |  | ✓ | ✓ | ✓ |
| Mantovani et al., 2017 **(109)** | African and African-Caribbean | ✓ | ✓ | ✓ | ✓ |  | ✓ | ✓ |  |  |
| Liljas et al., 2019 **(98)** | Socioeconomically deprived |  |  | ✓ |  |  | ✓ |  | ✓ |  |
| Gunner et al., 2019 **(95)** | Homeless and socioeconomically deprived | ✓ | ✓ |  | ✓ | ✓ | ✓ |  |  | ✓ |
| Wildman et al., 2019 **(101)** | Socioeconomically deprived |  | ✓ | ✓ |  |  | ✓ | ✓ | ✓ |  |
| Condon et al., 2020 **(116)** | Migrant parents and children | ✓ |  | ✓ |  | ✓ |  | ✓ |  |  |
| Hammad et al., 2020 **(102)** | Ethnic minority |  | ✓ | ✓ |  | ✓ | ✓ | ✓ |  | ✓ |
| Latif et al., 2020 **(97)** | Medically under-served including disabilities, homeless, BAME backgrounds, mental health conditions, or undergoing gender transition | ✓ | ✓ | ✓ |  |  | ✓ | ✓ |  |  |
| Linney et al., 2020 **(106)** | Somali migrant |  | ✓ | ✓ |  | ✓ | ✓ |  |  |  |
| Tomkow et al., 2020 **(90)** | Asylum seekers and refugees | ✓ | ✓ |  |  | ✓ |  |  |  |  |
| Woof et al., 2020 **(87)** | British-Pakistani and socioeconomically deprived | ✓ | ✓ | ✓ |  |  |  |  |  |  |
| Nellums et al., 2021 **(112)** | Undocumented migrants | ✓ |  |  | ✓ |  | ✓ |  | ✓ |  |
| Cook et al., 2021 **(117)** | Socioeconomically deprived |  |  |  |  |  | ✓ | ✓ |  | ✓ |
| Rayment-Jones et al., 2021 **(89)** | Ethnic minority | ✓ |  | ✓ |  |  | ✓ |  | ✓ |  |
| Anderson et al., 2022 **(82)** | Homeless, poor physical and mental health | ✓ | ✓ |  |  | ✓ |  | ✓ | ✓ |  |
| Smith, D. M. 2023 **(111)** | Socioeconomically deprived | ✓ | ✓ |  | ✓ | ✓ | ✓ |  |  |  |
| **The below articles collected data during and post-COVID-19** | | | | | | | | | | |
| * Heaslip et al., 2022 **(94)** | Homeless | ✓ | ✓ | ✓ | ✓ |  | ✓ |  |  |  |
| * Holding et al., 2022 **(99)** | Socioeconomically deprived |  | ✓ |  | ✓ | ✓ | ✓ |  |  | ✓ |
| * Thomson et al., 2022 **(110)** | Ethnic minority | ✓ |  | ✓ | ✓ |  | ✓ | ✓ |  |  |
| * Peñuela-O’Brien et al., 2023 **(118)** | Central and Eastern European migrant |  | ✓ | ✓ |  |  | ✓ | ✓ |  |  |
| * Crawshaw et al., 2023 **(107)** | Congolese migrant |  |  | ✓ |  |  | ✓ |  |  |  |
| * Rowe et al., 2023 **(100)** | Asylum seeking women | ✓ | ✓ | ✓ | ✓ |  |  | ✓ |  |  |
| * Jackson et al., 2024 **(113)** | Heavy alcohol use and poor mental health |  | ✓ |  | ✓ | ✓ | ✓ | ✓ |  | ✓ |
| * Smith et al., 2024 **(83)** | Socioeconomically deprived |  |  |  |  |  | ✓ |  |  |  |
| * The study collected data during or post-COVID-19 | | | | | | | | | | |
