# Supplementary material for: Perceived barriers and facilitators of accessing statutory and non-statutory services, in disadvantaged communities, in England: a co-produced qualitative review
Source: Public Health Rev. 2026 May 28;47:1608969. doi: 10.3389/phrs.2026.1608969 (PMC13377981; doi:10.3389/phrs.2026.1608969)
Supplement: Supplementary file 4 [file Supplementaryfile2.docx]

**Table 2. Critical Appraisal Skills Programme quality assessment (England, 2003–2024)**

| **Authors and Year - Data collected Pre-COVID-19** | **Was there a clear statement of the aims of the research?** | **Is a qualitative methodology appropriate?** | **Was the research design appropriate to address the aims of the research?** | **Was the recruitment strategy appropriate to the aims of the research?** | **Was the data collected in a way that addressed the research issue?** | **Has the relationship between researcher and participants been adequately considered?** | **Have ethical issues been taken into** | **Was the data analysis sufficiently rigorous?** | **Is there a clear statement of findings?** | **How valuable is the research?** | **Number of "Yes"es** |
| --- | --- | --- | --- | --- | --- | --- | --- | --- | --- | --- | --- |
| Jayaweera et al., 2005 **(104)** | Yes | Yes | Yes | Yes | Yes | No | No | No | Yes | Yes | 7 |
| Higginbottom, 2006 **(105)** | Yes | Yes | Yes | Yes | Yes | Not Met | Yes | Can`t tell | Not Met | Yes | 7 |
| Roddy et al., 2006 **(85)** | Yes | Yes | Yes | Yes | Yes | No | Yes | Yes | Yes | Yes | 9 |
| Moffatt et al., 2009 **(84)** | Yes | Yes | Yes | Yes | Yes | Yes | Yes | Can`t tell | Can`t tell | Yes | 8 |
| Coles et al., 2010 **(119)** | Yes | Yes | Yes | Yes | Yes | No | Yes | Yes | Yes | Yes | 9 |
| Ingram et al., 2010 **(122)** | Yes | Yes | Yes | Yes | Yes | No | Yes | Yes | Yes | Yes | 9 |
| Baxter et al., 2011 **(91)** | Yes | Yes | Yes | Yes | Yes | No | Yes | Can`t tell | Can`t tell | Yes | 7 |
| Leite et al., 2011 **(93)** | Yes | Yes | Yes | Yes | Yes | Can`t tell | Yes | Yes | Can`t tell | Yes | 8 |
| Williams et al., 2012 **(120)** | Yes | Yes | No | Yes | Yes | Yes | Yes | No | Yes | Yes | 8 |
| Newbigging et al., 2013 **(96)** | Yes | Yes | Yes | Yes | Yes | No | Yes | Yes | Yes | Yes | 9 |
| Ochieng, B. M. N. 2013 **(88)** | Yes | Yes | Yes | Yes | Yes | No | Yes | Yes | Can`t tell | Yes | 8 |
| Blickem et al., 2013 **(103)** | Yes | Yes | Yes | Yes | Yes | No | Yes | Yes | Can`t tell | Yes | 8 |
| Haddrill et al., 2014 **(121)** | No | Yes | No | No | Yes | No | Yes | No | Yes | Yes | 5 |
| Bains et al., 2015 **(115)** | Yes | Yes | Yes | Yes | Yes | No | Yes | Yes | Yes | Yes | 9 |
| Mastrocola et al., 2015 **(114)** | Yes | Yes | Yes | Yes | Yes | No | Yes | No | Yes | Yes | 8 |
| Memon et al., 2016 **(30)** | Yes | Yes | Yes | Yes | Yes | No | Yes | Yes | Can`t tell | Yes | 8 |
| Islam, M. P. 2016 **(92)** | Yes | Yes | Yes | Yes | Yes | No | Yes | Yes | Yes | Yes | 9 |
| Nyashanu et al., 2016 **(108)** | Yes | Yes | Yes | Yes | Yes | Yes | Yes | Yes | Can`t tell | Yes | 9 |
| Dharni et al., 2017 **(86)** | Yes | Yes | Yes | Yes | Yes | No | Yes | Yes | Can`t tell | Yes | 8 |
| Mantovani et al., 2017 **(109)** | Yes | Yes | Yes | Yes | Yes | Can`t tell | Can`t tell | Yes | Yes | Yes | 8 |
| Liljas et al., 2019 **(98)** | Yes | Yes | Yes | Yes | Yes | No | Yes | Yes | Yes | Yes | 9 |
| Gunner et al., 2019 **(95)** | Yes | Yes | Yes | Yes | Yes | No | Yes | Yes | Yes | Yes | 9 |
| Wildman et al., 2019 **(101)** | Yes | Yes | Yes | Yes | Yes | No | Yes | Yes | Yes | Yes | 9 |
| Condon et al., 2020 **(116)** | Yes | Yes | Yes | Yes | Yes | No | Yes | Yes | Yes | Yes | 9 |
| Hammad et al., 2020 **(102)** | Yes | Yes | Yes | Yes | Yes | No | Yes | No | Yes | No | 7 |
| Latif et al., 2020 **(97)** | Yes | Yes | Yes | Yes | Yes | No | No | Yes | Yes | Yes | 8 |
| Linney et al., 2020 **(106)** | Yes | Yes | Yes | Yes | Yes | Yes | Yes | Yes | Yes | Yes | 10 |
| Tomkow et al., 2020 **(90)** | Yes | Yes | Yes | Yes | Yes | Can`t tell | Yes | Can`t tell | Can`t tell | Yes | 7 |
| Woof et al., 2020 **(87)** | Yes | Yes | Yes | Yes | Yes | No | Yes | Yes | Can`t tell | Yes | 8 |
| Nellums et al., 2021 **(112)** | Yes | Yes | Yes | Yes | Yes | Can`t tell | Yes | Yes | Yes | Yes | 9 |
| Cook et al., 2021 **(117)** | Yes | Yes | Yes | Yes | Yes | No | Yes | Yes | Yes | Yes | 9 |
| Rayment-Jones et al., 2021 **(89)** | Yes | Yes | Yes | Yes | Yes | Yes | Yes | Yes | Yes | Yes | 10 |
| Anderson et al., 2022 **(82)** | Yes | Yes | Yes | Yes | Yes | No | Yes | Yes | Yes | Yes | 9 |
| Smith, D. M. 2023 **(111)** | Yes | Yes | Yes | Yes | Yes | Yes | Yes | Yes | Yes | Yes | 10 |
| **The below articles collected data during and post-COVID-19** | | | | | | | | | | | |
| * Heaslip et al., 2022 **(94)** | Yes | Yes | Yes | Yes | Yes | No | Yes | Yes | Yes | Yes | 9 |
| * Holding et al., 2022 **(99)** | Yes | Yes | Yes | Yes | Yes | No | Yes | Yes | Yes | Yes | 9 |
| * Thomson et al., 2022 **(110)** | Yes | Yes | Yes | Yes | Yes | No | Yes | Yes | Yes | Yes | 9 |
| * Peñuela-O’Brien et al., 2023 **(118)** | Yes | Yes | Yes | Yes | Yes | Yes | Yes | Yes | Can`t tell | Yes | 9 |
| * Crawshaw et al., 2023 **(107)** | Yes | Yes | Yes | Yes | Yes | Yes | Yes | Yes | Yes | Yes | 10 |
| * Rowe et al., 2023 **(100)** | Yes | Yes | Yes | Yes | Yes | Yes | Yes | Yes | Yes | Yes | 10 |
| * Jackson et al., 2024 **(113)** | Yes | Yes | Yes | No | No | No | Yes | No | No | Yes | 5 |
| * Smith et al., 2024 **(83)** | Yes | Yes | Yes | Yes | Yes | No | Yes | Yes | Yes | Yes | 9 |
| * The study collected data during or post-COVID-19 | | | | | | | | | | | |
