# Supplementary material for: Perceived barriers and facilitators of accessing statutory and non-statutory services, in disadvantaged communities, in England: a co-produced qualitative review
Source: Public Health Rev. 2026 May 28;47:1608969. doi: 10.3389/phrs.2026.1608969 (PMC13377981; doi:10.3389/phrs.2026.1608969)
Supplement: Supplementary file 5 [file Supplementaryfile4.docx]

**Table 4. Study and sample characteristics of the articles included in the review (England, 2003–2024)**

| **Authors and Year - Data collected Pre-COVID-19** | **Region of England** | **Service focus** | **Location of Data collection** | **Sample size and Gender distribution** | **Age Summary** | **Health characteristics of the populations(s)** | **Data analysis** | **The key findings of the study** |
| --- | --- | --- | --- | --- | --- | --- | --- | --- |
| Jayaweera et al., 2005 **(104)** | Leeds (North) | Maternity services | Homes of participants | *N* = 9 (9 females) | *M* = 24 years, *SD* = Unspecified, Range: 20 years-30 years | Pregnant or new mothers (recent childbirth) | Theme-based qualitative analysis | Bangladeshi mothers faced language barriers, poor treatment, and limited culturally appropriate maternity care |
| Higginbottom, 2006 **(105)** | Sheffield (North) and Nottingham (East) | Hypertension management in African Caribbean patients | GP surgeries and health centres | *N* = 36 (Unspecified) | *M* = 59.5 years, *SD* = Unspecified, Range: 37 years-82 years | Hypertension | A focused ethnography which draws on the adjectival tradition | Cultural beliefs, poor communication, and mistrust reduced African Caribbean use of GP services |
| Roddy et al., 2006 **(85)** | Nottingham (East) | Smoking cessation services | Community-based smoking cessation services and local premises for focus groups​ | *N* = 39 (16 females, 23 males) | *M* = 45 years (Median), *SD* = Unspecified, Range: 27 years-77 years | Smoking addiction, high nicotine dependence, previous unsuccessful quit attempts, perceived stress-related smoking, concerns about weight gain after quitting, withdrawal symptoms from previous quit attempts, psychological dependency on smoking, feelings of marginalisation and stigma related to smoking | Grounded theory approach with iterative thematic/category development | Deprived smokers avoided services due to stigma, misinformation, and lack of tailored support |
| Moffatt et al., 2009 **(84)** | Newcastle upon Tyne (Northeast) | Welfare rights advice for older South Asians | Community events, participants' homes, GP practices, welfare rights service offices, minority ethnic community support service resource centres | *N* = 22 (Unspecified) | *M* = Unspecified, *SD* = Unspecified, Range: 50 years-81 years | Multiple chronic health problems, diabetes, cardiovascular disease, joint problems | Framework method/ analysis | Respectful, home-based advice helped older South Asians access welfare they previously avoided |
| Coles et al., 2010 **(119)** | Halton and St Helens (Northwest) | Community-based men’s health promotion service | Community venues | *N* = 82 (82 males) | *M* = Unspecified, *SD* = Unspecified, Range: 40 years-Early 80s | Stress, isolation, long-term illness, ageing-related health problems, heart attack, testicular cancer, fear, embarrassment, reluctance to seek help, lack of health awareness | Framework analysis | Men avoided health services due to fear, pride, and feeling unwelcome or judged |
| Ingram et al., 2010 **(122)** | Bristol (Southwest) | School-based sexual health drop-in service | School-based clinics, pupil referral units, music rooms, empty classrooms, counselling rooms within schools | *N* = 44 (Unspecified) | *M* = 14.7 years, SD = Unspecified, Range: 11 years-20 years | Early sexual activity, teenage pregnancy, sexually transmitted infections (STIs), unprotected sex, coercion or pressure to have sex, relationship issues, low academic attainment, school exclusion | Thematic analysis | School clinics reached vulnerable youth early, but cultural stigma and visibility deterred some groups |
| Baxter et al., 2011 **(91)** | York (North) | Health services for disabled adults and older people with support services on decision-making about care options, and information accessibility | Hospitals, GP surgeries, social care services, community organisations, people's homes | *N* = 50 (Unspecified) | *M* = Unspecified, SD = Unspecified, Range: 25 years-65 years+ | Fluctuating support needs, sudden onset of support needs, arthritis, multiple sclerosis | Theme-based qualitative analysis | People with gradual needs missed services due to poor, late, or absent information |
| Leite et al., 2011 **(93)** | Unspecified | Healthcare and social care support for people with chronic fatigue syndrome | Community settings, GP surgeries, patients' homes, hospitals | *N* = 35 (26 females, 9 males) | *M* = Unspecified, *SD* = Unspecified, Range: 18 years-56 years+ | Chronic fatigue syndrome (CFS/ME), anxiety, depression, severe muscular pain, physical and mental fatigue, neurological symptoms, gastrointestinal symptoms, cardiovascular issues, social exclusion, difficulty with daily activities | Inductive thematic analysis | People with chronic fatigue syndrome needed belief, practical help, and protection from stigma |
| Williams et al., 2012 **(120)** | Birmingham (West) | Preventative, community-based child and family health service | Children’s centre, cafés, church meeting rooms, participants' homes, community centres, health centres | *N* = 46 (46 males) | *M* = Unspecified, *SD* = Unspecified, Range: 18 years-Unspecified | Hypertension, diabetes, obesity, smoking, alcohol use, stress, tiredness, concern for children’s health and well-being, reluctance to use health services, fear of surveillance or misdiagnosis, experiences of racism, stigma, discrimination | Abductive reasoning and thematic categorisation | Fathers prioritised children’s health but felt excluded by services and misunderstood by professionals |
| Newbigging et al., 2013 **(96)** | Manchester and Preston (Northwest) | Mental health advocacy and community-based support, empowerment, and advocacy models for African and Caribbean men | Community organisations, mental health advocacy services, hospitals, GP surgeries, social care services, voluntary sector organisations | *N* = 25 (25 males) | *M* = Unspecified, *SD* = Unspecified, Range: Early 20s-Mid-40s | Serious mental illness, schizophrenia, depression, dual diagnosis (mental illness and substance misuse) | Systematic thematic analysis | Culturally aware, independent advocates helped African Caribbean men navigate mental health systems |
| Ochieng, B. M. N. 2013 **(88)** | Unspecified (North) | Health promotion services for Black African migrants | Local health centres and GP practices | *N* = 90 (50 females, 40 males) | *M* = Unspecified, *SD* = Unspecified, Range: 18 years-65 years | Anxiety, emotional distress, pregnancy-related health concerns | A variation of the constant comparative method | Language barriers and poor cultural fit blocked Black African migrants from using health promotion |
| Blickem et al., 2013 **(103)** | Greater Manchester (Northwest) | Long-term condition (LTC) support utilising social prescribing, voluntary, and community support networks to improve well-being | Community centres, and support group locations | *N* = 37 (Unspecified) | *M* = Unspecified, *SD* = Unspecified, Range: 50s-70s | Diabetes, cardiovascular disease, mobility issues, social isolation and loneliness | Thematic analysis | Supportive link workers helped people with long-term conditions join local health activities |
| Haddrill et al., 2014 **(121)** | Sheffield (North) | Antenatal care | Homes, community children's centres, hospital antenatal clinics, hospital antenatal wards | *N* = 27 (27 females) | *M* = 26 years, *SD* = Unspecified, Range: 15 years-37 years | Learning disabilities, substance misuse, mental health struggles (including fear, anxiety, depression, ambivalence), previous traumatic childbirth, unplanned/unwanted pregnancy, previous concealed pregnancy, poor reproductive health knowledge, belief in subfertility (e.g. due to age, past illness, or contraceptive use) | Iterative thematic analysis | Delayed antenatal care stemmed from denial, stigma, service confusion, and negative past experiences |
| Bains et al., 2015 **(115)** | Nottingham (East) | Community-based mobile stop smoking service | Supermarket car parks, leisure centre car parks, community centre car parks, industrial estates | *N* = 36 (25 females, 11 males) | *M* = 40 years, *SD* = Unspecified, Range: 17 years-70 years | Asthma, angina, stress, depression, nicotine dependence, weight gain, cravings, irritability, difficulty stopping smoking | Framework approach | Mobile services helped deprived smokers quit by being convenient, informal, and instantly accessible |
| Mastrocola et al., 2015 **(114)** | Unspecified (Northwest) | Long-term condition management for women involved in street-based prostitution | Inside the Manchester Action on Street Health (MASH) building, a third-sector support organisation | *N* = 16 (16 females) | *M* = 38.5 years, *SD* = Unspecified, Range: 22 years-60 years | Asthma, Emphysema, Recurrent chest infections, Recurrent bronchitis, Chronic deep vein thrombosis (DVT), Aortic and tricuspid regurgitation (secondary to endocarditis), Osteoarthritis, Hypertension, Chronic lower back pain, Rheumatoid arthritis, Diabetes, ‘Fits’, ‘Heart problems’, Hepatitis C, Cardiovascular accident (CVA), Depression, Anxiety, Psychosis, Paranoia, Bipolar disorder, Alcohol dependency, Stress. | Inductive analysis using constant comparison, with an approach derived from framework analysis | Street-based women delayed care due to stigma, homelessness, and negative GP experiences |
| Memon et al., 2016 **(30)** | Brighton and Hove (South) | Mental health services for BME communities | Community organisations, mental health services, GP surgeries, hospitals | *N* = 26 (13 females, 13 males) | *M* = Unspecified, *SD* = Unspecified, Range: 18 years-65 years | Inability to recognise mental health problems, psychological distress, anxiety, depression, psychotic disorders | Thematic analysis | BME people avoided mental health services due to racism, shame, and cultural misunderstandings |
| Islam, M. P. 2016 **(92)** | Bradford (North) | Breastfeeding peer support programme | Children’s centres, homes, infant feeding cafés, postnatal wards, community venues | *N* = 11 (11 females) | *M* = 29 years, *SD* = Unspecified, Range: 16 years-42 years | Mental health issues, depression, social isolation, substance misuse, low confidence, previous negative experiences with health services | Thematic content analysis | Distrust, lack of relevance, and poor timing deterred disadvantaged women from breastfeeding support |
| Nyashanu et al., 2016 **(108)** | Birmingham, Coventry, and Wolverhampton (West) | HIV prevention and sexual health services for Black sub-Saharan African communities | Community groups and local sexual health organisations | *N* = Unclear (Unspecified) | *M* = Unspecified, *SD* = Unspecified, Range: 16 years-60 years | HIV/AIDS, Sexually Transmitted Infections | A qualitative analysis using the Silences Framework | Stigma, mistrust, and cultural clashes blocked HIV prevention in African communities |
| Dharni et al., 2017 **(86)** | Lambeth and Southwark in Southeast London (South) | Colorectal cancer screening | GP surgeries | *N* = 50 (21 females, 29 males) | *M* = 65.61 years (Men), 65.13 years (Women), *SD* = 4.73 years (Men), 4.54 years (Women), Range: 55 years-74 years | Colorectal cancer, previous cancer experience, family history of cancer, prostate cancer awareness, physical/mobility limitations, existing physical or mental health problems | Framework Analysis, with the Theoretical Domains Framework (TDF) | Screening was avoided due to anxiety, shame, and confusing test procedures |
| Mantovani et al., 2017 **(109)** | South London (South) | Mental health services with community-based support, social engagement, and well-being initiatives | Faith-based organisations (FBOs) and community organisations (COs) | *N* = 13 (6 females, 7 males) | *M* = 49 years, *SD* = Unspecified, Range: 24 years-75 years | Mental health issues, stress and emotional distress, depression, fear and stigma related to mental health, lack of knowledge about mental health | A qualitative participatory evaluation using iterative thematic/analytical framework approach | Community-led, culturally grounded approaches improved mental health engagement among African populations |
| Liljas et al., 2019 **(98)** | North London (South) | Health promotion and engagement strategies, social determinants, and support for hard-to-reach older adults | Primary care practices, a Black and Minority Ethnic (BME) community group, and a day centre for older people | *N* = 19 (Unspecified) | *M* = Unspecified, *SD* = Unspecified, Range: 65 years-85 years | Hypertension, multiple chronic conditions, mobility problems, sensory impairments (vision and hearing loss), memory problems, dementia | Thematic Framework analysis | Engagement improved when health promotion respected older people’s identities, autonomy, and local knowledge |
| Gunner et al., 2019 **(95)** | West Midlands | Primary healthcare services for people who are homeless | Homeless shelters, specialist primary healthcare centre for homeless people, GP surgeries, hospitals | *N* = 22 (5 females, 15 males, 2 unspecified) | *M* = Unspecified, *SD* = Unspecified, Range: 24 years-70 years | Chronic health conditions, comorbidities, epilepsy, diabetes, infectious diseases (HIV, hepatitis C), mental health conditions, substance misuse | Thematic framework approach | Homeless individuals experience systemic barriers to mainstream care but value accessible, integrated specialist services |
| Wildman et al., 2019 **(101)** | Newcastle upon Tyne (Northeast) | Social prescribing services for individuals with long-term health conditions | GP surgeries, community hubs, patients' homes, voluntary sector organisations | *N* = 24 (11 females, 13 males) | *M* = Unspecified, *SD* = Unspecified, Range: 40 years-74 years | Diabetes (types 1 and 2), chronic obstructive pulmonary disease (COPD), asthma, coronary heart disease, heart failure, epilepsy, osteoporosis, anxiety, depression, social isolation, multimorbidity | Thematic analysis, within a grounded theory approach | Link worker social prescribing builds confidence and connection, helping people manage health and life challenges |
| Condon et al., 2020 **(116)** | Unspecified (Southwest) | Child health services for migrant parents with social support-based involvement in parenting and migrant family support | Health visitors conducted child health promotion services in family homes | *N* = 28 (22 females, 6 males) | *M* = 30 years-35 years (by group), *SD* = Unspecified, Range: 17 years-47 years | Obesity, Dental caries (tooth decay), infectious diseases | Thematic content analysis | Mistrust, stigma, and spiritual beliefs shape Somali reluctance to seek formal mental health support |
| Hammad et al., 2020 **(102)** | London (South) | Hand of Hope culturally appropriate, faith-informed, trauma-informed, coproduced, therapeutic group intervention | Community (retail) pharmacies, drug treatment services locations | *N* = 10 (10 females) | *M* = Unspecified, *SD* = Unspecified, Range: 36 years-83 years | Posttraumatic stress disorder (PTSD), traumatic bereavement, low mood, social isolation | Thematic analysis | Culturally grounded, faith-sensitive therapy fostered trust and healing in Muslim communities post-Grenfell fire |
| Latif et al., 2020 **(97)** | East Midlands | Pharmacy medication reviews for marginalised patient groups | Community pharmacies and patients' homes​ | *N* = 20 (10 females, 10 males) | *M* = Unspecified, *SD* = Unspecified, Range: 19 years-67 years | Disabilities (DEAF, blind, physical), neurodegenerative disorders, multiple morbidities, ill mental health, substance abuse | Interpretivist, framework-informed coding analysis using iterative coding | Marginalised patients mistrust, misunderstand, and struggle to access medicine reviews. |
| Linney et al., 2020 **(106)** | Bristol (Southwest) | Mental health services for Somali communities | Community-based participatory research sites, likely including Somali community centres and outreach locations | *N* = 23 (Unspecified) | *M* = Unspecified, *SD* = Unspecified, Range: Late 20s-60s | Headaches, depression, anxiety, post-traumatic stress disorder (PTSD), suicidal thoughts or self-harm | Thematic analysis, using constant comparison | Somali views link mental illness to stigma and spirituality, limiting access to formal mental healthcare |
| Tomkow et al., 2020 **(90)** | Manchester (Northwest) | Primary care with community-based support for asylum seekers and refugees | Voluntary Community Organizations (VCOs) | *N* = 18 (11 females, 7 males) | *M* = Unspecified, *SD* = Unspecified, Range: 18 years-47 years | Mental health conditions (e g, depression), chronic lung disease, dental problems, physical pain and mobility issues, vitamin D deficiency, obesity and overweight, cancer history, pregnancy and maternity care needs, need for emergency healthcare services, general health deterioration after arrival in the UK, experience of trauma and post-migration stress | Thematic analysis | Confusion among migrants and staff about healthcare eligibility blocks safe, timely NHS access |
| Woof et al., 2020 **(87)** | East Lancashire (Northwest) | Breast cancer screening services for British-Pakistani women | NHS breast screening clinics and community outreach events | *N* = 19 (19 females) | *M* = Unspecified, *SD* = Unspecified, Range: Under 50 years-50 years+ | Breast cancer, obesity | Thematic analysis, using a realist and manifest-inductive approach | Culturally tailored, community-based communication can improve breast screening access for British-Pakistani women |
| Nellums et al., 2021 **(112)** | East London (South) | Maternity services for undocumented migrant women | Doctors of the World (DOTW) clinic | *N* = 20 (20 females) | *M* = Unspecified, *SD* = Unspecified, Range: 21 years-50 years | Pregnancy complications, premature birth, unexpected caesarean, financial stress impacting health, mental health stress, limited access to antenatal care | Thematic analysis | Charging policies compromise care quality and safety for undocumented women during NHS maternity treatment |
| Cook et al., 2021 **(117)** | Luton (East) | Breastfeeding support services | Community centres | *N* = 63 (63 females) | *M* = Unspecified, *SD* = Unspecified, Range: 21 years-45 years | Pain during breastfeeding, perceived lack of milk supply, mental health stress, mastitis, low birth weight infants, postnatal depression | Framework Analysis | Breastfeeding support must be culturally sensitive, accessible, and ongoing to meet diverse mothers’ needs |
| Rayment-Jones et al., 2021 **(89)** | Unspecified | Interpreter services, within maternity services, for pregnant women | Hospitals, maternity clinics, GP surgeries, community-based maternity services | *N* = 8 (8 females) | *M* = Unspecified, *SD* = Unspecified, Range: 18 years-34 years+ | Mental health issues, domestic violence, previous sexual abuse/trafficking, female genital mutilation (FGM), pregnancy-related complications | Thematic framework analysis | Relational, consistent interpreting improves safety, trust, and care for pregnant women with language barriers |
| Anderson et al., 2022 **(82)** | Bristol (Southwest) | Physical health service for people who inject drugs | A nurse-led walk-in service embedded within the Bristol Drugs Project (BDP) building | *N* = 9 (Unspecified) | *M* = Unspecified, *SD* = Unspecified, Range: 18 years-Unspecified | Substance use disorders and related infections | Combined deductive framework analysis and inductive thematic analysis | Non-judgmental, flexible, and co-located care makes physical health services acceptable to people who inject drugs |
| Smith, D. M. 2023 **(111)** | Medway Towns (Southeast of London, Southeast) | Community health and safety services in low-income neighbourhoods | Community centres, residents' homes, schools, churches, and social events | *N* = 18 (11 females, 7 males) | *M* = Unspecified, *SD* = Unspecified, Range: 21 years-55 years | Poor mental health | Theme-based qualitative analysis using constant comparative assessment | Austerity eroded public services, worsening health and safety, prompting grassroots community-led responses |
| **The below articles collected data during and post-COVID-19** | | | | | | | | |
| * Heaslip et al., 2022 **(94)** | Poole, Bournemouth, and Boscombe (Southwest) | Access to health services and support services such as food banks, shelters, and financial assistance | Community centres, GP surgeries, homeless shelters, night shelters, churches, food banks, internet cafés, libraries | *N* = 16 (4 females, 12 males) | *M* = 41.48 years (All), 41.46 years (Males), 41.50 years (Females), *SD* = 11.72 years (All), 11.13 years (Males), 14.12 years (Females), Range: All: 20 years-71 years, Males: 20 years-66 years, Females: 22 years-71 years | Gastric issues, addiction, anxiety, depression, mental health issues, dietary issues, physical health issues | Inductive thematic analysis | Homeless people rely on word-of-mouth and trusted relationships to discover health and social care services |
| * Holding et al., 2022 **(99)** | South Yorkshire (North) and London (South) | Mental health services with support from community-based youth organisations providing informal mental health support for young people | Youth organisations | *N* = 42 (18 females, 1 gender fluid, 19 males, 2 non-binary, 2 trans males) | *M* = 16.7 years, *SD* = Unspecified, Range: 13 years-21 years | Mental health issues, social anxiety, exam stress, peer pressure, bullying, low self-esteem, body image concerns, chronic stress, depression, suicidal thoughts | Thematic analysis | Young people want accessible, non-judgmental, and proactive mental health support tailored to their realities |
| * Thomson et al., 2022 **(110)** | Unspecified (Northwest) | Maternity services and perinatal mental health services for minoritised ethnic women | Hospitals, GP surgeries, community organisations, maternity clinics, neonatal units, women's homes | *N* = 23 (23 females) | *M* = Unspecified, *SD* = Unspecified, Range: 20 years-50 years | Fluctuating support needs, mental health conditions, antenatal depression, postnatal depression, anxiety, post-traumatic stress disorder (PTSD), pregnancy-related complications, perinatal loss | Thematic analysis | Relational, respectful care from trusted staff mitigates discrimination and improves experiences for ethnic minority women |
| * Peñuela-O’Brien et al., 2023 **(118)** | Greater Manchester (Northwest) | Mental health services for Central and Eastern European migrants | NHS mental health services, university counselling services, third-sector organisations, private therapy, and workplace counselling services | *N* = 13 (9 females, 4 males) | *M* = Unspecified, *SD* = Unspecified, Range: 18 years-59 years | Mild to moderate depression, anxiety, severe depression, psychotic episode | Reflexive thematic analysis, conducted inductively | Cultural stigma, invisibility, and language barriers limit Central and Eastern European migrants’ access to effective mental healthcare |
| * Crawshaw et al., 2023 **(107)** | Hackney (South) | Vaccination uptake among Congolese migrants | Community venues in Hackney, London, including community centres, refugee and migrant forums, and support group spaces | *N* = 32 (24 females, 8 males) | *M* = 52.6 years, *SD* = 11.0 years, Range: 25 years-65 years+ | Blood clot concerns, polio-related disability, fear of long COVID, negative side effects from previous vaccine doses | Thematic analysis, with findings then mapped to the Theoretical Domains Framework (TDF) and COM-B model for intervention development | Community co-designed, culturally tailored interventions can effectively address vaccine hesitancy among Congolese migrants |
| * Rowe et al., 2023 **(100)** | Liverpool (Northwest) | Maternal health services for asylum-seeking women, and support with the asylum-seeking process, and social service referrals | Hospitals, GP surgeries, community organisations, women's homes, drop-in centres | *N* = 16 (Unspecified (women only)) | *M* = 33 years (Median), *SD* = Unspecified, Range: 24 years-47 years | Fluctuating support needs, psychological stress, anxiety, depression, suicidal ideation, self-harm, hypertension, iron deficiency anaemia, deep vein thrombosis, low platelet count, panic attacks | Thematic framework analysis | Asylum-seeking women with multimorbidity face compounding health, social, and system barriers to safe maternity care |
| * Jackson et al., 2024 **(113)** | Newcastle upon Tyne (Northeast) | Integrated mental health and substance use care service | Private rooms in community venues | *N* = 39 (18 females, 21 males) | *M* = Unspecified, *SD* = Unspecified, Range: 20 years-69 years | Heavy alcohol use, depression, suicidal thoughts, self-harm, mental ill-health, substance use, physical health conditions, economic deprivation, social isolation, digital exclusion | Inductive coding, constant comparison, with relational autonomy | People facing disadvantage value relational, navigable care over fragmented, bureaucratic health and social systems |
| * Smith et al., 2024 **(83)** | Yorkshire (North) | Smoking cessation support in lung screening participants | Lung screening vans (mobile units) and telephone-based support​ | *N* = 30 (15 females, 15 males) | *M* = 69 years, *SD* = Unspecified, Range: 56 years-79 years | Smoking addiction, high nicotine dependence, chronic obstructive pulmonary disease (COPD), sleep apnea, back pain (without a diagnosed underlying cause), undiagnosed depression, undiagnosed anxiety, perceived stress-related smoking, low motivation to quit smoking, social isolation-related distress, psychological dependency on smoking | Thematic analysis, using both inductive and deductive approaches | Psychosocial barriers and low motivation deterred smokers from engaging with cessation support during screening |
| * The study collected data during or post-COVID-19 | | | | | | | | |
